# Optimized bacterial DNA isolation method for microbiome analysis of human tissues

**DOI:** 10.1002/mbo3.1191

**Published:** 2021-06-16

**Authors:** Carlijn E. Bruggeling, Daniel R. Garza, Soumia Achouiti, Wouter Mes, Bas E. Dutilh, Annemarie Boleij

**Affiliations:** ^1^ Department of Pathology Radboud Institute for Molecular Life Sciences (RIMLS) Radboud University Medical Center (Radboudumc) Nijmegen The Netherlands; ^2^ Radboud Institute for Molecular Life Sciences (RIMLS) Center for Molecular and Biomolecular Informatics (CMBI) Radboud University Medical Center (Radboudumc) Nijmegen The Netherlands; ^3^ KU Leuven Department of Microbiology, Immunology and Transplantation Laboratory of Molecular Bacteriology Rega Institute Leuven Belgium; ^4^ Department of Animal Ecology & Physiology Institute for Water and Wetland Research (IWWR) Radboud University Nijmegen The Netherlands; ^5^ Department of Microbiology Institute for Water and Wetland Research (IWWR) Radboud University Nijmegen The Netherlands; ^6^ Theoretical Biology and Bioinformatics Science for Life Utrecht University Utrecht The Netherlands

**Keywords:** bacterial DNA enrichment, tissue microbiome profiling

## Abstract

Recent advances in microbiome sequencing have rendered new insights into the role of the microbiome in human health with potential clinical implications. Unfortunately, the presence of host DNA in tissue isolates has hampered the analysis of host‐associated bacteria. Here, we present a DNA isolation protocol for tissue, optimized on biopsies from resected human colons (~2–5 mm in size), which includes reduction of human DNA without distortion of relative bacterial abundance at the phylum level. We evaluated which concentrations of Triton and saponin lyse human cells and leave bacterial cells intact, in combination with DNAse treatment to deplete released human DNA. Saponin at a concentration of 0.0125% in PBS lysed host cells, resulting in a 4.5‐fold enrichment of bacterial DNA while preserving the relative abundance of *Firmicutes*, *Bacteroidetes*, *γ*‐*Proteobacteria*, and *Actinobacteria* assessed by qPCR. Our optimized protocol was validated in the setting of two large clinical studies on 521 in vivo acquired colon biopsies of 226 patients using shotgun metagenomics. The resulting bacterial profiles exhibited alpha and beta diversities that are similar to the diversities found by 16S rRNA amplicon sequencing. A direct comparison between shotgun metagenomics and 16S rRNA amplicon sequencing of 15 forceps tissue biopsies showed similar bacterial profiles and a similar Shannon diversity index between the sequencing methods. Hereby, we present the first protocol for enriching bacterial DNA from tissue biopsies that allows efficient isolation of all bacteria. Our protocol facilitates analysis of a wide spectrum of bacteria of clinical tissue samples improving their applicability for microbiome research.

## Graphical abstract

We present a bacterial DNA isolation method for human colon biopsies which includes reduction of human DNA and does not distort relative bacterial abundance at the phylum level. Shotgun sequencing resulted in 508/521 metagenomes (Shannon index; 2.9, UniFrac distance; 0.56). Fifteen colon biopsies were sequenced by both amplicon and shotgun sequencing resulting in similar bacterial profiles from phylum to genus level. Our protocol facilitates analysis of a wide spectrum of bacteria of clinical tissue samples improving their applicability for microbiome research.

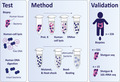

## 1 INTRODUCTION

The rapidly growing field of microbiome research is steadily revealing the role of the microbiome in human health and diseases. Functions of the gut microbiome are diverse and essential for many biological processes involved in metabolism, tissue homeostasis, and immunity (Lynch & Pedersen, 2016). Changes in microbiome composition have been associated with a wide variety of diseases, ranging from intestinal inflammatory diseases to colorectal cancer to diseases outside the gastrointestinal tract (Lynch & Pedersen, 2016). Such compositional changes are well‐studied by microbiome profiling through the sequencing of DNA isolates. While a vast amount of research has been performed on stool, recent technologies have facilitated bacterial profiling on colon tissues, which allows more localized analysis (Saffarian et al., 2019) and may be more accurate in differentiating between healthy and diseased states (Bajaj et al., 2012). Importantly, DNA isolation methods have a major impact on the evaluation of microbiota composition (Bajaj et al., 2012; Hasan et al., 2016; Knudsen et al., 2016; Lim et al., 2018; Nelson et al., 2019; Thoendel et al., 2016; Wagner Mackenzie et al., 2015; Wesolowska‐Andersen et al., 2014; Yuan et al., 2012). Hence, a well‐developed and standardized protocol for stool and tissues will contribute to consensus in microbiome research.

The study of microbiome composition of solid tissue samples, however, does not come without challenges. Whole tissue isolates contain large bulks of host DNA, overshadowing the presence of single‐cell organisms and viruses. While polymerase chain reaction (PCR) is a valuable technique to identify minority sequences, the field of microbiome research is slowly moving toward shotgun metagenomic sequencing as a preferred method. Shotgun metagenomic sequencing allows analysis of all sequences in the DNA isolate, resulting in an increased species detection with higher accuracy (Ranjan et al., 2016). Another major advantage of this technique is the ability to discriminate between microbial species and analyze their gene content including potential virulence factors (Ranjan et al., 2016). This may be crucial to discriminate between a pathogen and a commensal bacterium at the species level (Taddese et al., 2019). Unfortunately, the application of shotgun metagenomic sequencing to study the microbiome of human tissue is severely limited by the high amount of human DNA present in these samples, which vastly outnumbers the bacterial DNA.

Various methods have been developed to improve the bacterial‐to‐human DNA ratio. These methods include filtering out human cells by size (Marotz et al., 2018), antibody‐mediated filtration of human DNA by targeting non‐methylated CpG dinucleotide motifs (Horz et al., 2010; Marotz et al., 2018), and human‐specific cell lysis followed by DNA degradation (Horz et al., 2010; Marotz et al., 2018; Nelson et al., 2019; Thoendel et al., 2016), of which the latter results in most efficient bacterial DNA enrichment (Marotz et al., 2018; Nelson et al., 2019). Bacterial DNA enrichment contributes to the identification of minority species and higher sequencing coverage of the microbial genomes present in human tissue samples, thus improving the taxonomic and functional analysis of the microbiome in these samples.

One of the caveats of bacterial DNA enrichment is that the method of DNA isolation affects the microbiome profile (Biesbroek et al., 2012; Bjerre et al., 2019; Horz et al., 2010; Knudsen et al., 2016; Marotz et al., 2018; Nelson et al., 2019; Thoendel et al., 2016). Bacteria differ in their susceptibility to lysis, resulting in the tendency of some bacteria to lyse too early during the isolation method (Biesbroek et al., 2012; Horz et al., 2010), while other bacteria may require extra steps to release their DNA, for example, by mechanical lysis through bead‐beating (Lim et al., 2018; Yu et al., 2017). The addition of mechanical lysis has improved the isolation of Gram‐positive bacteria (Biesbroek et al., 2012; Knudsen et al., 2016; Yuan et al., 2012), without impairing the isolation of Gram‐negative bacteria (de Boer et al., 2010). Additionally, enzymatic lysis with mutanolysin may enrich for Gram‐positive bacteria (Moen et al., 2016; Yuan et al., 2012). The ultimate goal of these strategies is to increase the bacterial‐to‐human DNA ratio and have a DNA isolate that closely reflects the bacterial composition of the sample.

The immense advance in our understanding of the human gut microbiome has been largely based on stool samples; not tissue. Thereby, the study of the bacteria that reside in closest proximity to the host has been largely neglected, along with crucial information about their localization in the gut (e.g., colonic segment or localization to tumors). To address the current limitations in obtaining bacterial DNA from gut tissue samples that is suitable for shotgun metagenomic sequencing, here we present an optimized DNA isolation method. Our method is modified from the HMP project (Gevers et al., 2012) and combines important elements of the currently best‐performing methods for DNA isolation, that is bacterial DNA enrichment, mutanolysin treatment, heat shock, and bead‐beating. Our protocol efficiently lyses Gram‐positive bacteria, while maintaining the DNA derived from the Gram‐negative bacteria. Our optimized protocol enriches the bacterial content of biopsies ranging from ~2–5 mm and was validated in the context of two large prospective studies on *in vivo* acquired tissue biopsies using shotgun metagenomics. This method will contribute to reproducible research in the field of bacterial microbiome composition and function and will be of value not only for gut‐related tissue but also for those tissues where bacteria are underrepresented.

## 2 METHODS

### 2.1 Collection of human colon biopsies

*Ex vivo* residual resected colon material was obtained at the department of pathology of the Radboudumc in Nijmegen between 2017 and 2018, in accordance with Dutch legislation. Twenty forceps biopsies of about 2 mm were taken from 2 resected colons (10 biopsies of patients 1 and 2) and 24 biopsies of about 5 mm were taken from 5 resected colons (4, 2, 8, 4, and 6 biopsies of patient 3–7 respectively). No approval from a research ethics committee was required for the study of residual colon resections because anonymous use of redundant tissue for research purposes is part of the standard treatment agreement with patients in the Radboudumc, to which patients may opt‐out. Resected colons were transported from the operation room to the Pathology suite, and tissue was rinsed with dH_2_O before taking samples in a clean and well‐ventilated non‐sterile environment. None of the included patients submitted an objection against the use of residual materials, and all material was processed anonymously. Biopsies were resected with a clean scalpel, resulting in biopsies up to an estimated size of 5 mm. Alternatively, biopsy forceps were used to make biopsies of about 2 mm that were used as a proxy for biopsies taken during colonoscopy. After collection, biopsies were snap‐frozen in cryo‐tubes in liquid nitrogen and stored at −80°C.

*In vivo* collected forceps biopsies for shotgun metagenomic sequencing were obtained from patients that came for a screening colonoscopy and participated in either of the two clinical prospective studies: the BBC study (NL57875.091.16), which involved solely genetically confirmed Lynch syndrome patients, or the BaCo study (NL55930.091.16), which included ulcerative colitis patients and patients without known colon diseases. Two healthy appearing tissue biopsies were taken with sterile forceps in colon ascendens (VR1) and descendens (VR2), with optional one extra biopsy in or close to suspected precancerous lesions or inflammation (VR3) and were collected immediately in sterile tubes in liquid nitrogen. All samples were collected between 2017 and 2018 in Radboudumc Nijmegen. Both studies were approved by the Internal Revenue Board CMO‐Arnhem Nijmegen (CMO 2016–2616 and CMO 2016–2818) and the board of the Radboudumc. Patients who had taken antibiotics within the last 3 months before the colonoscopy were excluded. All patients were older than 18 years and signed informed consent. Biopsies were snap‐frozen in cryo‐tubes in liquid nitrogen instantly after collection and stored at −80°C. For an overview of the study steps, patients, and biopsies used for each analysis, see Table A1 in Appendix 1.

### 2.2 Bacterial DNA isolation protocol

**FIGURE 1:**
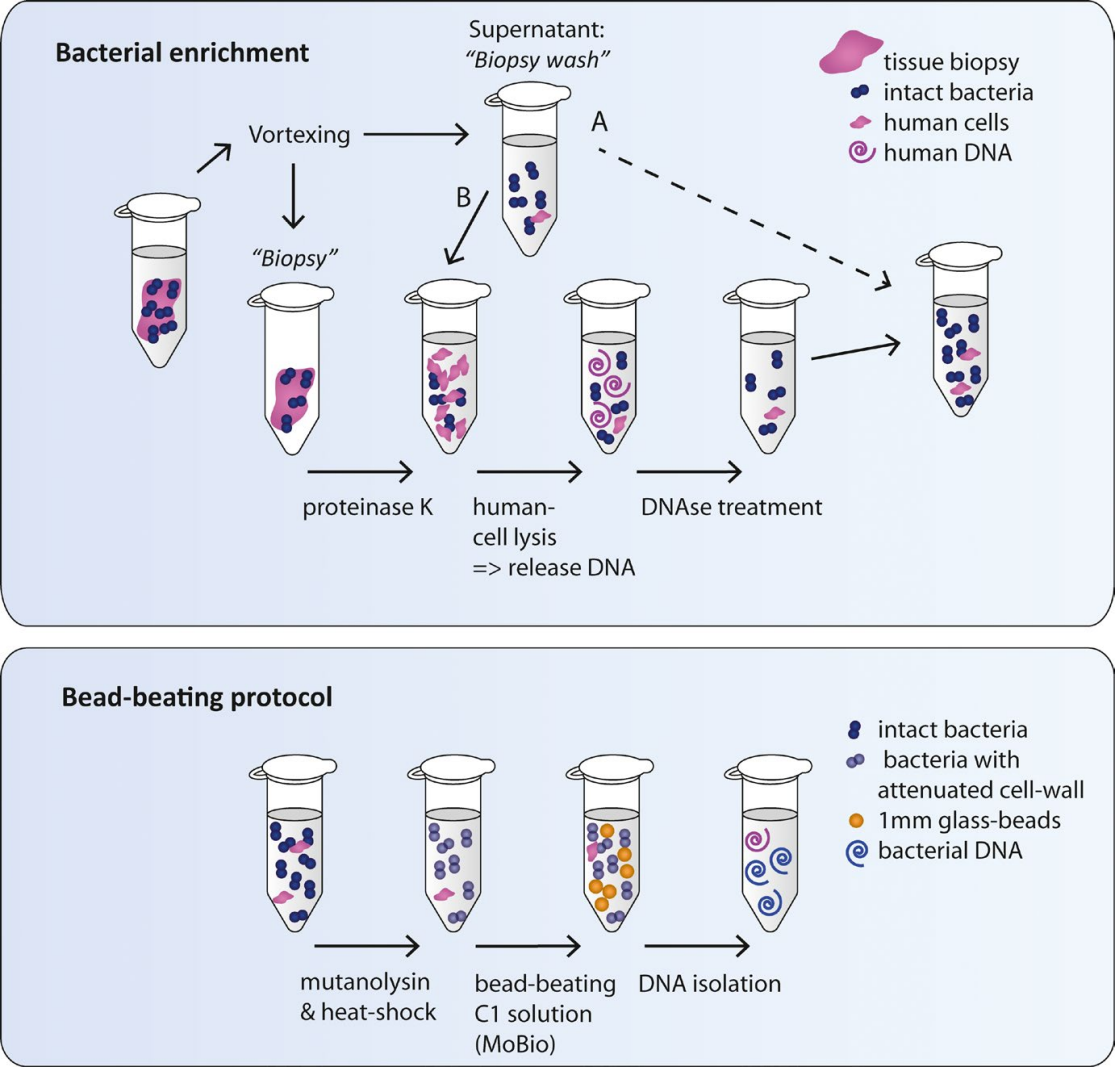
Schematic drawing of DNA isolation protocol strategy 2. (a) Bacterial enrichment: A tissue biopsy is vortexed in PBS to separate bacteria from the biopsy. The biopsy is retrieved for digestion with proteinase K, while the supernatant (biopsy wash) is saved on ice and added back for DNA isolation at a later timepoint (timepoint A or B; B in the final protocol). Bacteria in the biopsy wash are thereby minimally exposed to reagents that could cause possible lysis; however, this suspension contains human cells and/or released human DNA and should therefore follow route B. Subsequently, 0.0125% saponin in PBS is added to the cell suspension inducing lysis of human cells, but not bacterial cells. DNA in the supernatant is depleted through DNAse treatment. The remaining sample has reduced human DNA content and still intact bacteria. (b) Bead‐beating protocol: The sample is further processed by our previously optimized bead‐beating protocol. Mutanolysin treatment followed by heat shock is applied to attenuate cell walls of Gram‐positive bacteria (e.g., *Streptococci* and *Actinobacteria*) to make them more susceptible for mechanical lysis. Subsequently, the sample is bead‐beaten with 1 mm glass beads in C1 buffer of the PowerLyzer PowerSoil DNA Isolation Kit and further isolated according to the manufacturer's protocol. The resulting DNA isolate is enriched for bacterial DNA

**FIGURE A1:**
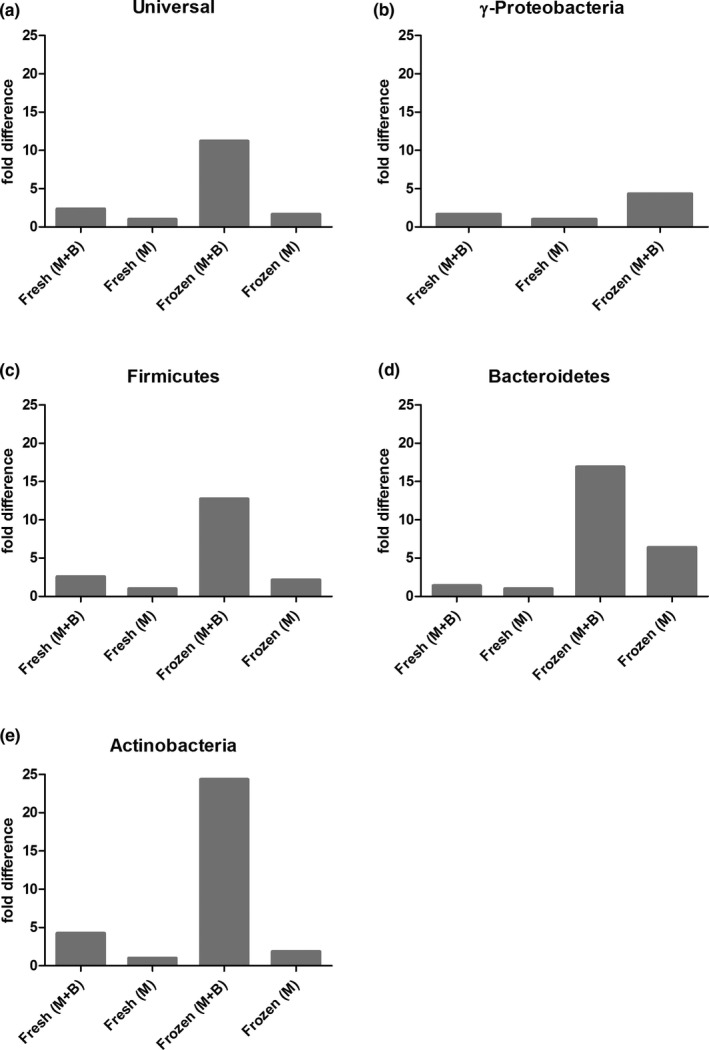
Ultra‐deep microbiome prep kit performs better on frozen tissue in combination with our optimized bead‐beating protocol. Healthy biopsies (~5 mm) from 1 patient were either snap‐frozen (frozen) or immediately isolated with the Ultra‐deep microbiome prep kit (fresh). Isolation was either performed with the full protocol provided by Molzym (M) or was combined with bead‐beating (M + B). The fold difference represents the bacterial signal relative to the positive control (feces) (ΔCt) and was compared to sample Fresh (M) (ΔΔCt)

The bacterial DNA isolation strategy involved bacterial DNA enrichment through human cell lysis and DNAse treatment (Figure 1, upper part), which was followed up by our previously optimized bead‐beating protocol (Figure 1, lower part) (Couto Furtado Albuquerque et al., 2017). Whereas the bead‐beating protocol remained unchanged throughout this paper, two alternative strategies were tested for bacterial DNA enrichment. For the first strategy, the Molzym DNA isolation (Ultra‐Deep Microbiome prep, Molzym, 2020) kit was used. The manufacturer's protocol was followed until and including the molDNAse inactivation step. Subsequently, the bead‐beating protocol was applied to assist in mechanical bacterial cell lysis, because this was shown to result in a higher bacterial signal in qPCR (Figure A1 in Appendix 2). For the second strategy, we established our alternative protocol including proteinase K (19133, Qiagen) for protein digestion, Phosphate‐buffered saline (PBS) (Braun, 220/12257974/1110) containing saponin (47036‐50G, Sigma‐Aldrich) or Triton (9002‐93‐1, Sigma‐Aldrich) for selective lysis of host cells, and TurboDNAse (AM2239, Qiagen) for host DNA removal. We evaluated the effect of detergents, Triton or saponin, at different concentrations for lyses of human cells and experimented what was the best moment to include the biopsy wash (point A or B) in the DNA isolation process (Figure 1).

The lysis of bacterial cells included treatment with 0.5 KU/mL mutanolysin (SAE0092, Sigma‐Aldrich), heat shock, and buffer C1 of the DNAeasy PowerLyzer PowerSoil kit from Qiagen (previously known as the MoBio PowerLyzer PowerSoil DNA isolation kit from MoBio). Bead‐beating was performed in the Magnalyser (Roche) at 6400 rpm for 20 s twice, with 30 s on ice in between. After bacterial lysis, the manual of the DNA isolation kit was followed. The final protocol is provided in Appendix 3. Our final bacterial enrichment protocol (Figure 1, route B and Appendix 3) was also tested by an independent laboratory (Institute for Water and Wetland Research, Radboud University) for isolation of bacteria from zebrafish gills, but in combination with CTAB extraction instead of the MoBio DNA isolation kit (Appendix 4).

### 2.3 Bacterial culturing

*Collinsella intestinalis* (DSM13280), *Bacteroides vulgatus* (3775 SL(B)10), *Escherichia coli* (NTB5), and *Streptococcus gallolyticus* subsp. *gallolyticus* (UCN34) were cultured on Brain Heart Infusion agar plates supplemented with yeast extract L‐cysteine Vitamin K, and Hemin (BHI‐S; ATCC medium 1293). *C*. *intestinalis* and *B*. *vulgatus* were grown on plates for 48 hr under anaerobic conditions before transfer to liquid medium for 48–72 hr at 37°C. *E*. *coli* and *S*. *gallolyticus* were grown overnight on plated under aerobic conditions before transfer to liquid culturing in BHI for 24 hr at 37°C/5% CO_2_. Bacteria were pelleted by centrifugation at 4600 rpm for 10 min and frozen at −20°C. Bacterial pellets were thawed and dissolved in PBS until 1 optical density (OD at 620 nm) of which 50 µl was used for experiments to determine bacterial DNA release by Triton and saponin treatment.

To create a mock community, 1 OD bacterial PBS suspensions were mixed in 400 µ​l (40% *B. vulgatus*, 30% *E. coli*, 20%, *S. gallolyticus*, and 10% *C. intestinalis*) and were pelleted for each experimental condition.

### 2.4 Bacterial DNA release by treatment with Triton and saponin

Bacteria were dissolved in PBS with final concentrations of the detergents Triton (%v/v) or saponin (%w/v) of 0.1%, 0.025%, 0.0125%, and 0.006%. Bacteria were incubated for 30 min at 37°C with detergent or PBS only. Samples were centrifuged at 10,000×*g* for 10 min, and the DNA concentration was measured with Qubit Fluorometer 2.0 (Thermo Fisher Scientific) using the Qubit dsDNA HS assay kit (Q32856, Thermo Fisher). A Mann–Whitney U test was used to compare the DNA in the supernatants of samples exposed to detergent versus PBS.

### 2.5 Effects of saponin 0.0125% on human tissue lysis

To test whether saponin 0.0125% was able to induce human cell lysis, resected human colon biopsies of an estimated size of 5 mm were processed according to our optimized protocol up to the step of selective cell lysis using saponin (Figure 1 and Appendix 3). During this last step, cell pellets were incubated with either 0.0125% saponin or PBS in turboDNAse buffer, but without turboDNAse enzyme. Samples were incubated at 37°C for 30 min to lyse the cells, and the supernatant was cleared from cell debris by two centrifugation cycles of 10 min at 10,000×*g* at 4°C. DNA in the supernatant was precipitated with 100% ethanol and centrifuged at 10,000×*g* at 4°C for 20 min. Precipitated DNA was washed with 70% ethanol and centrifuged at 10,000×*g* at 4°C for 20 min. Lastly, DNA was air‐dried and resuspended dH_2_O.

### 2.6 Quantitative Real‐Time PCRs for 16S rRNA

Each reaction for qPCR consisted of 0.4 µM forward primer, 0.4 µM reverse primer, 1X Power SYBR Green (A4368702, Applied biosystems). The amount of DNA in each reaction was 1 ng and 0.1 ng for biopsies that were ~5 mm and ~2 mm, respectively. Primers for the host (human or zebrafish) and bacteria (all bacteria, *Firmicutes*, *Bacteroidetes*, *γ*‐*Proteobacteria*, and *Actinobacteria*) were used and evaluated previously (Albuquerque et al., 2017; Bacchetti De Gregoris et al., 2011; Yang et al., 2015) and are reported in Table A2 in Appendix 1 (Amann et al., 1990; Bacchetti De Gregoris et al., 2011; Silva et al., 2009; Gorissen et al., 2009; Juretschko et al., 1998; Yang et al., 2015). qPCRs were performed with a 7500 Fast Real‐Time PCR system (Applied Biosystems®). Samples were heated to 50°C for 2 min, 95°C for 10 min, 30 cycles of 95°C for 15 s and 60°C for 1 min, followed by a continuous sequence of 95°C for 15 s, 60°C for 1 min, 95°C for 30 s, and 60°C for 15 s. Melting curves were generated to evaluate the specificity of the PCR product. All qPCR analyses were performed in duplicate.

DNA isolated from the mock community (described above) was used as a positive control. Only for Figure A1 in Appendix 2, a human fecal reference isolate was used as a calibrator sample for relative abundance. Reference DNA isolated from human blood served as a negative control to set background qPCR signals.

### 2.7 Statistical analysis of qPCRs

To evaluate differences in bacterial content between samples, the universal 16S rRNA signal of the sample was calibrated using the universal 16S rRNA signal of the positive control (ΔCt); a mock community isolate. Fold difference was calculated by 2^−ΔCt^. Metagenomic analysis revealed that the most common phyla were Firmicutes (39.8%), Bacteroidetes (16.7%), Actinobacteria (9.3%), Proteobacteria (16.4%), Verrocumicrobia (0.2%) and others (17.5%) (Figure 4c). Subsequently, the ΔCt was compared to the ΔCt in a control sample (ΔΔCt). Fold difference was calculated by 2^−ΔΔCt^. Paired samples were analyzed with a paired t‐test. In the case of unmatched samples, the Mann–Whitney U test was used for comparison. A Friedman test was used to evaluate which detergent resulted in the most similar bacterial composition to PBS. All statistical tests were performed using Graphpad Prism version 5.0.

### 2.8 Shotgun metagenomic sequencing of human in vivo acquired colon biopsies

DNA was isolated using our optimized protocol including the DNeasy Powerlyzer Powersoil kit (Qiagen), as described in Appendix 3. DNA concentration was measured as described previously. A total of 521 human colon tissue DNA isolates from 226 patients were sent to Novogene Bioinformatics Technology Co., Ltd in Hong Kong for sequencing. Samples were processed using low input NEBnext library preparation, and paired‐end sequencing was performed on the Illumina Novaseq 6000 with 350 bp insert size and a read length of 150 bp. 1.2 GB output data in FastQ format were guaranteed per sample. Samples were measured for DNA concentration (Qubit), and construct length and a quality check were performed on the library preparation. Thirteen samples were not sequenced due to failed library preparation resulting in 508 successfully sequenced metagenomes of 224 patients (Supplementary Data S1: https://doi.org/10.5281/zenodo.4678214).

In addition, for the comparison of 16S rRNA versus metagenomics sequencing, the second set of 15 biopsy samples of 12 patients were selected from the BBC study that had the highest DNA yields. These samples had an average concentration of 5.9 ng/µl. 5 µl was used for 16S rRNA amplification, while the rest for metagenomics library preparation. The samples were sent to Novogene Bioinformatics Technology Co., Ltd in Hong Kong for sequencing. Metagenomics sequencing was performed as described above. The V3‐V4 region of the 16S rRNA gene was amplified using primer 341F (CCTAYGGGRBGCASCAG) and 806R (GGACTACNNGGGTATCTAAT). All PCR reactions were carried out with Phusion® High‐Fidelity PCR Master Mix (New England Biolabs). The libraries were generated with NEBNext® UltraTM DNA Library Prep Kit for Illumina and quantified via Qubit and qPCR. Sequencing was performed on Illumina NovaSeq 6000 platform to generate 250 bp paired‐end raw reads (Q30 > 94.8%) (Supplementary Data S2: https://doi.org/10.5281/zenodo.4678214).

### 2.9 Bioinformatics analysis

Quality control, trimming, and removal of adaptors were performed using FastQC version 0.11.9 and trimmomatic version 0.35. An assembly dataset was generated by filtering out the human reads using BBMap version 38.84 with the GRCh38 version of the human genome. Filtered reads were assembled with metaSPAdes version 3.13.1. The taxonomic classification of contigs was determined with CAT v. 4.6 (von Meijenfeldt et al., 2019) using the NCBI NR as a database for taxonomic assignments. bwa version 0.7.17 and samtools version 1.9 were used to map all the reads to the classified contigs and the human genome and to estimate the coverage statistics. For the analysis in Figure 4c+d, only the samples with more than 2.0e04 bacterial reads were used, resulting in 379/508 (74.6%) metagenomes derived from human colon biopsies (belonging to 203 of 224 patients) with an average of 11 million reads per sample. This cutoff was used to guarantee the generation of reliable profiles from bacterial reads (Cattonaro et al., 2018; Louca et al., 2018; Zeller et al., 2014). Since this cutoff was determined artificially, we repeated the same analysis with the full dataset (Figure A6a+b in Appendix 2). Samples were rarified by resampling the reads according to the samples with the fewest number of reads. Shannon diversity (alpha) and the UniFrac diversity (beta)(Lozupone & Knight, 2005) were estimated from the taxonomic distribution of reads at the genus level. Diversity indices and phylum level classifications were compared to values obtained from literature selected based on sequencing of colon tissue biopsies reporting Shannon diversity and phylum abundance. We did not perform a meta‐analysis and also did not download the raw data, but used the reported metrics as a comparison for our metagenome results. Studies fulfilling these criteria were 16S rRNA amplicon‐based (Djuric et al., 2019; Kiely et al., 2018; Momozawa et al., 2011; Watt et al., 2016). In addition, we performed a direct comparison between 16S rRNA sequencing and shotgun metagenomics for 15 samples. The shotgun metagenomic samples were processed as described above. The paired‐end reads generated from 16S rRNA sequencing were assigned to samples based on their unique barcodes and truncated by cutting off the barcode and primer sequences. Paired‐end reads were merged using FLASH (V1.2.7; Magoc & Salzberg, 2011). Quality filtering on the raw tags was performed under specific filtering conditions to obtain high‐quality clean tags (Bokulich et al., 2013) according to the Qiime (V1.7.0) quality‐controlled process (Caporaso et al., 2010). The tags were compared with the reference database using the UCHIME algorithm to detect chimera sequences (Edgar et al., 2011), which were subsequently removed to obtain effective tags. Sequence analyses were performed by Uparse software (Edgar, 2013) using all the effective tags. Sequences with ≥97% similarity were assigned to the same OTUs. For each representative sequence, Mothur software was performed against the SSUrRNA database of the SILVA Database (Wang et al., 2007) for species annotation at each taxonomic rank (Threshold:0.8~1) (Quast et al., 2013). The OTUs abundance information was normalized using a standardized sequence number corresponding to the sample with the least sequences. Subsequent analysis of Shannon index 2.9 and UniFrac distance 0.56 was all performed on these normalized data and compared to those obtained from shotgun metagenomics (Supplementary Data S2: https://doi.org/10.5281/zenodo.4678214).

## 3 RESULTS

### 3.1 Whole tissue digestion including PBS wash is required to capture the collective tissue microbiome

**FIGURE A2:**
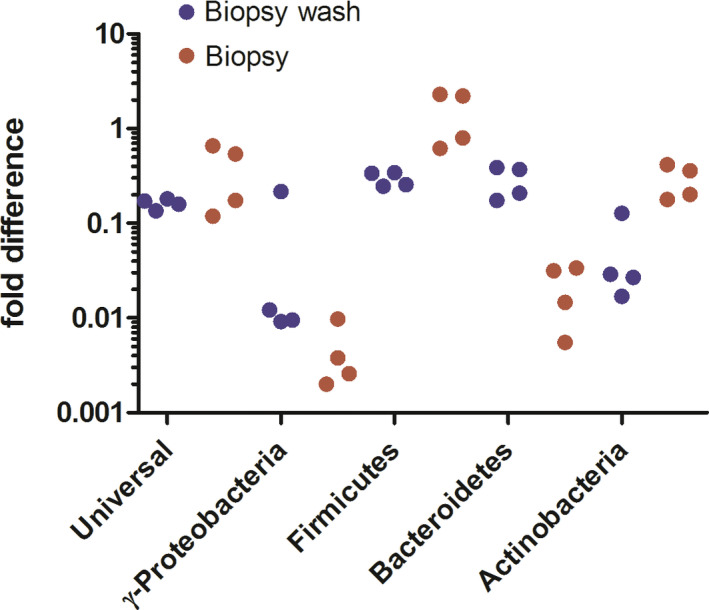
Whole tissue digestion is required to isolate all bacteria. Two matched biopsies (~5 mm) were washed in PBS, after which DNA of the Biopsy wash and the Biopsy was isolated separately. For every DNA isolate, a duplicate was run, of which each value is plotted relative to the mock community (ΔCt). Paired t‐tests revealed that DNA from the biopsy isolates contained a similar bacterial fraction, albeit with fewer *Bacteroidetes* and more *Actinobacteria*. Hence, whole tissue digestion is required to analyze the complete bacterial component of the tissue

Because a commercial kit (Molzym, 2020) was available to enrich bacterial DNA, we started by testing this method. In addition, because it is hypothesized that the major bulk of human DNA in the microbial DNA isolate could be avoided by only isolating DNA from washed tissue (biopsy wash), we tested whether the biopsy wash only would be sufficient for bacterial analysis. To test this, the biopsy and biopsy wash were isolated separately with the Ultra‐Deep Microbiome prep kit (Molzym, 2020) in combination with our bead‐beating protocol. While biopsies were isolated with the full protocol including protein digestion, selective lysis, and removal of human DNA using strategy 1 (see *Methods*), these steps were omitted for the biopsy wash (Figure 1, path A). Similar universal bacterial 16S rRNA signals were obtained from DNA isolates of the biopsy wash and biopsies (Figure A2 in Appendix 2).

**FIGURE A3:**
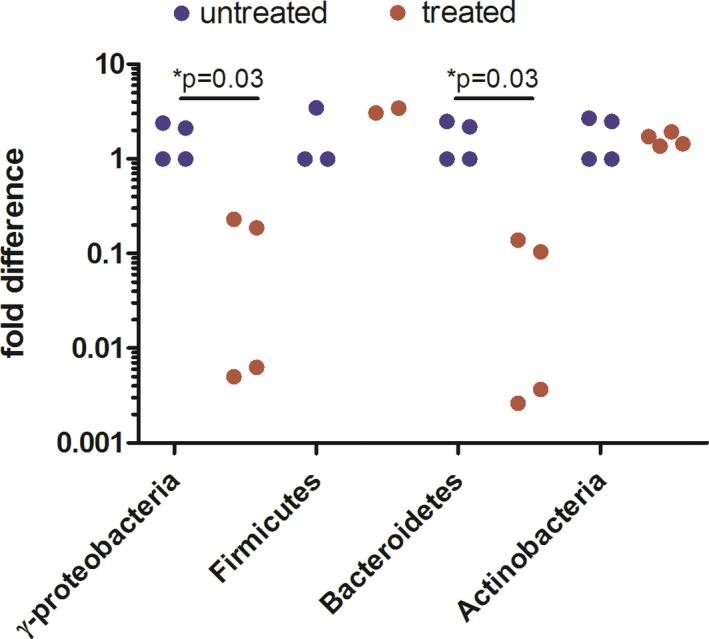
Ultra‐Deep Microbiome prep on bacterial mock community results in the underrepresentation of *γ*‐*Proteobacteria* and *Bacteroidetes*. Two bacterial pellets (mock community) were isolated with the full protocol (treated), whereas 2 pellets were isolated skipping proteinase K, mild lysis, and DNAse treatment (untreated). To investigate alterations in bacterial composition, each sample was calibrated with its own universal 16S rRNA signal (ΔCt) and was compared to one untreated sample (ΔΔCt). Each sample was run as a PCR duplicate of which both data points were plotted. Mann–Whitney t‐test revealed a significant decrease compared to PBS for *γ*‐*Proteobacteria* and *Bacteroidetes*

Interestingly, the biopsy wash appeared to have relatively more Gram‐positive and fewer Gram‐negative bacteria compared to the microbiota remaining in the matched biopsy, although this was not significant (Figure A2 in Appendix 2). Therefore, we tested the effect of strategy 1 on a mock community by comparing the full protocol (similarly to the biopsy) to a part of the protocol (similarly to the biopsy wash, Path A in Figure 1). We found that the full strategy 1 protocol, which includes selective cell lysis and DNAse treatment, resulted on average in a 15‐fold lower signal of *γ‐Proteobacteria* (*p* = 0.03) and a 27‐fold lower signal of *Bacteroidetes* (*p* = 0.03) as opposed to the incomplete protocol (Figure A3 in Appendix 2). Although only tested on the mock community, this result was for us unacceptable to continue strategy 1 as it suggests that it disfavors isolation of Gram‐negative bacteria versus Gram‐positive bacteria.

### 3.2 Saponin 0.0125% seems safe to use to lyse host cells, but not bacterial cells

**FIGURE 2:**
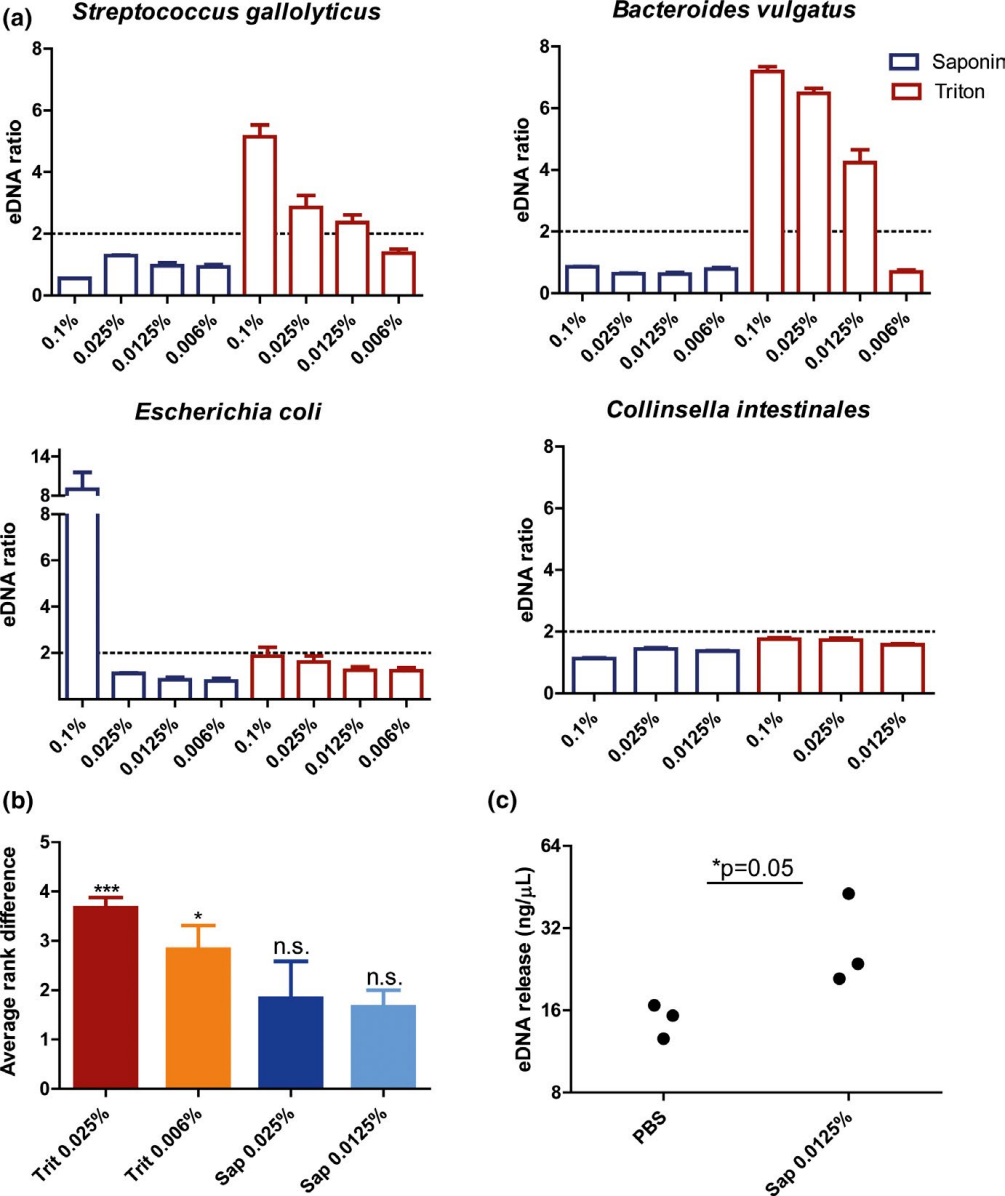
(a) Saponin 0.0125% induces human cell lysis, without inducing bacterial cell lysis. The effect of Triton and saponin on bacterial cell lysis was measured. This experiment was performed for *Streptococcus gallolyticus* *(Firmicutes)*, *Bacteroides vulgatus* (*Bacteroidetes*), *Escherichia coli* (*γ*‐*Proteobacteria*), and *Collinsella intestinales* (*Actinobacteria*). The ratio between the concentration in treated versus untreated (PBS) was plotted. An increase of more than 2 was considered relevant. Results show that Triton affects bacterial cell lysis in *Streptococcus gallolyticus* and *Bacteroides vulgatus*, but not in *Escherichia coli* and *Collinsella intestinalis*. Saponin only induced cell lysis at 0.1% in *E. coli*. (b) Biopsies were isolated with strategy 2 in combination with Triton (Trit) and saponin (Sap) at different concentrations. The relative bacterial signal for *Firmicutes*, *Bacteroidetes*, *Actinobacteria*, and *γ*‐*Proteobacteria* was calibrated with the universal 16S rRNA signal (ΔCt) and was compared to PBS (ΔΔCt). Similarity to PBS was calculated through ranking using the Friedman test. Both saponin concentrations most closely resembled bacterial composition in PBS and hence preserved bacterial composition at phylum level in the colon biopsies. (c) DNA release of biopsies was measured after exposure to either PBS or saponin 0.0125%. More external DNA (eDNA) was measured after incubation with saponin 0.0125% (*p* = 0.05), suggesting that human cell lysis was induced, although eDNA was also detected in the sample with PBS alone

Strategy 2 was established using similar, but tweakable steps, including protein digestion with proteinase K, selective human cell lysis with detergents, and DNAse treatment to remove host cell DNA after lysis. First, we tested which detergent would effectively lyse human cells without affecting the ratio of bacterial phyla. Hence, we tested whether treatment with different concentrations of Triton and saponin would result in bacterial DNA release (eDNA) of pure cultures and affected bacterial phyla in tissue biopsies compared to PBS. First, pure bacterial cultures of *Streptococcus gallolyticus* *(Firmicutes)*, *Bacteroides vulgatus (Bacteroidetes)*, *Escherichia coli (γ*‐*Proteobacteria)*, and *Collinsella intestinalis* *(Actinobacteria)* (Figure 2a) were exposed to Triton and saponin. While *C*. *intestinalis* was resistant to lysis under all conditions, *B*. *vulgatus* and *S*. *gallolyticus* were susceptible to lysis in the presence of Triton, with higher concentrations leading to more eDNA. Triton did not affect the amount of eDNA of *E*. *coli* and *C*. *intestinalis*. Saponin was shown to be a mild detergent, as it only increased the eDNA of *E*. *coli* at a concentration of 0.1%. These experiments suggest that saponin concentrations equal to or lower than 0.025% and Triton concentrations equal to or lower than 0.006% are safe for bacterial lysis.

**FIGURE A4:**
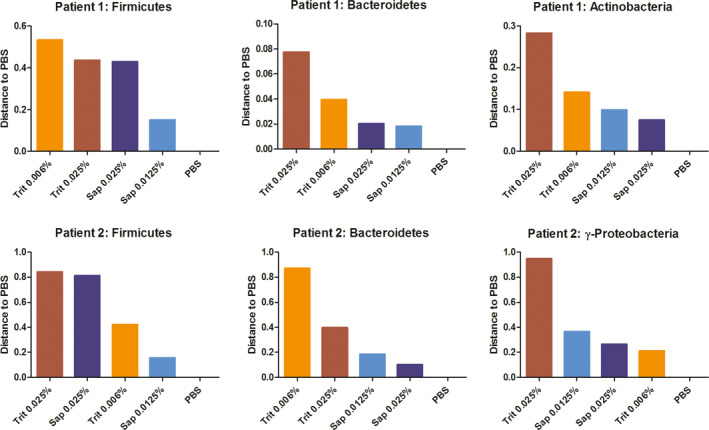
Effect of detergent on bacterial composition. Colonic biopsies (~3 mm) from 2 patients were isolated with our protocol using different detergents and concentrations. The bacterial signal for *Firmicutes*, *Bacteroidetes*, *Actinobacteria*, and *γ*‐*Proteobacteria* was calibrated with the universal 16S rRNA signal of the same patient (ΔCt) and was compared to PBS sample of the same patient (ΔΔCt). The difference to PBS was plotted

**FIGURE A5:**
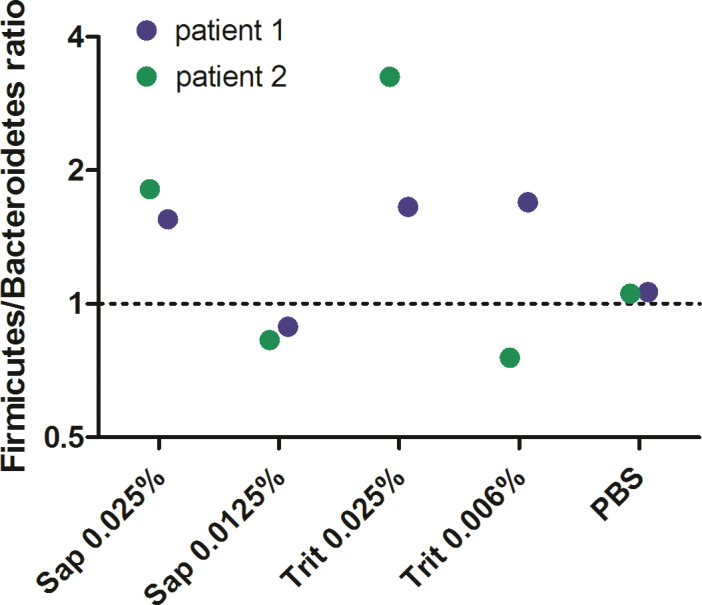
*Firmicutes* to *Bacteroidetes* ratio is least affected by saponin 0.0125%. This graph is extracted from the same experiment as represented in Figure A4 in Appendix 2. For both *Bacteroidetes* and *Firmicutes*, the signal was calibrated with the positive control (mock community) (ΔCt). The enrichment ratio was calculated by 2^−ΔCt^(*Firmicutes*)/2^−ΔCt^(*Bacteroidetes*)

Secondly, it was tested whether Triton and saponin would change the bacterial composition of 20 matched tissue biopsies at phyla level from 2 patients (patient 1 and patient 2). DNA was isolated using the protocol including either saponin (0.0125% or 0.025%) or Triton (0.025% or 0.006%) and the relative abundance of *Firmicutes*, *Bacteroidetes*, *Actinobacteria*, and *γ*‐*Proteobacteria* was compared to isolations performed without detergents (PBS). For each phylum, the detergent creating the lowest distance to PBS was ranked 1, followed by rank 2, 3, and 4 (Figure A4 in Appendix 2). Saponin 0.0125% led to the smallest difference in abundance with PBS across all bacterial phyla (Figure 2b). Triton 0.006% and Triton 0.025% ranked significantly higher (*p* < 0.05 and *p* < 0.001 respectively) (Figure 2b). Additionally, the *Firmicutes* to *Bacteroidetes* ratio was only maintained in the saponin 0.0125% condition (Figure A5 in Appendix 2). Thus, saponin 0.0125% preserved relative bacterial composition at phyla level within the samples and seems safe to use to lyse host cells.

Thirdly, we tested whether saponin 0.0125% would mediate human cell lysis by exposing 2 sets of 3 tissue homogenates (size: ~5 mm; step after biopsy proteinase K treatment in (Figure 1)) to either PBS or saponin 0.0125%. The supernatant of the tissues treated with saponin contained more than twice the amount of eDNA compared to tissues in PBS only (*p* = 0.05) (Figure 2c). This shows that exposure of tissue to saponin 0.0125% induces lysis of host cells.

### 3.3 Strategy 2 increases the bacterial‐to‐human signal

**FIGURE 3:**
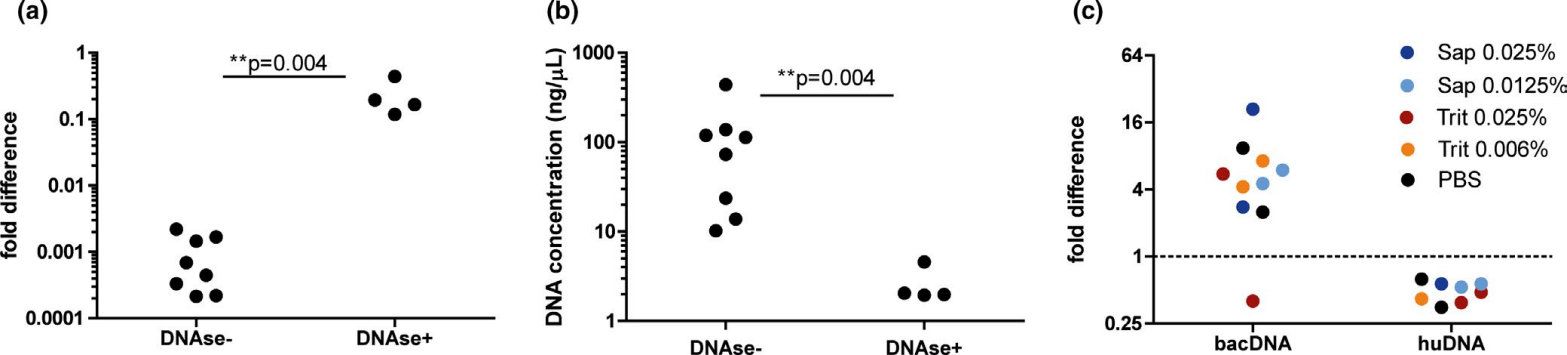
DNAse treatment lowers total DNA yield and improves bacterial‐to‐human DNA signal. **(**a + b) To test the effectiveness of bacterial DNA enrichment, isolations were performed on tissues (~5 mm) with or without the biopsy wash included in the DNAse treatment (DNAse+and DNAse‐ respectively, which represent path b and a respectively in Figure 1). DNAse treatment results higher bacterial signal (*p* = 0.004) (a) which corresponds with a lower DNA yield (*p* = 0.004) (b). These results suggest that DNAse treatment on the PBS wash enriches the bacterial DNA content of the isolate, illustrating that PBS wash should be included during DNAse treatment (path B in Figure 1). (c) To test the effect of enrichment on small‐sized biopsies, 5 pairs of forceps biopsies were taken from resected colons of 2 patients. Each pair was isolated with a different detergent condition of which 1 sample was isolated with DNAse and the other without. The fold difference of bacterial 16S rRNA signal (bacDNA) and human KRAS signal (huDNA) between these samples (ΔCt) is plotted (10 data points, 5 for each patient). DNAse treatment resulted in a 1.9‐fold reduction of human DNA signal (huDNA ratio 0.53, CI: 0.42–0.65). The bacterial signal was enriched 6.8‐fold on average (CI: 2.2–10.52) upon DNA treatment. Triton 0.006% and saponin 0.0125% with DNAse rendered more than 4.3 and 4.5‐fold increased bacterial signal respectively in both patients

After DNA release of human tissue, DNAse treatment should be performed to degrade the released DNA. Degradation of eDNA significantly reduced free DNA in the supernatant (Figure 3b). The significantly lower DNA yield after DNAse treatment was associated with an increased bacterial signal in qPCR (*p* = 0.004) (Figure 3a), which is indicative of a greater bacterial‐to‐human DNA fraction in the tissue DNA isolate and suggests bacterial DNA enrichment.

Next, we validated our protocol on biopsies from resected colons, which were taken using forceps to represent clinical biopsies taken during colonoscopy (size: ~2 mm). 5 pairs of biopsies were taken from 2 different patients. Each biopsy pair was isolated with the same detergent concentrations, of which only one was treated with DNAse. DNAse treatment reduced the human signal in qPCR to 0.53 (CI:0.42–0.65) but increased the bacterial signal 6.8‐fold (CI: 2.2–10.52) (Figure 3c). Triton 0.006% and saponin 0.0125% gave an enrichment of greater than 4 in both patients (Figure 3c). Interestingly, also in absence of detergent (PBS control), DNAse treatment resulted in bacterial signal enrichment. This could be explained by the presence of human eDNA due to human cell lysis that may occur during repetitive heating and centrifugation. Ultimately, the bacterial enrichment protocol of strategy 2 was applied in an independent laboratory to isolate bacterial DNA from fish gills. Use of saponin 0.0125% and DNAse treatment doubled the bacterial in qPCR and reduced host signal by factor 135 times, indicating that our enrichment protocol is reproducible and applicable for a wider variety of tissues (Table A3 in Appendix 1).

Taken together, our results show that strategy 2, including host cell lysis with 0.0125% saponin and DNAse treatment, successfully decreases human DNA in the sample and boosts the bacterial signal.

### 3.4 The bacterial composition of human colon tissue biopsies by shotgun metagenomics resembles that previously reported by 16S rRNA analysis

**FIGURE 4:**
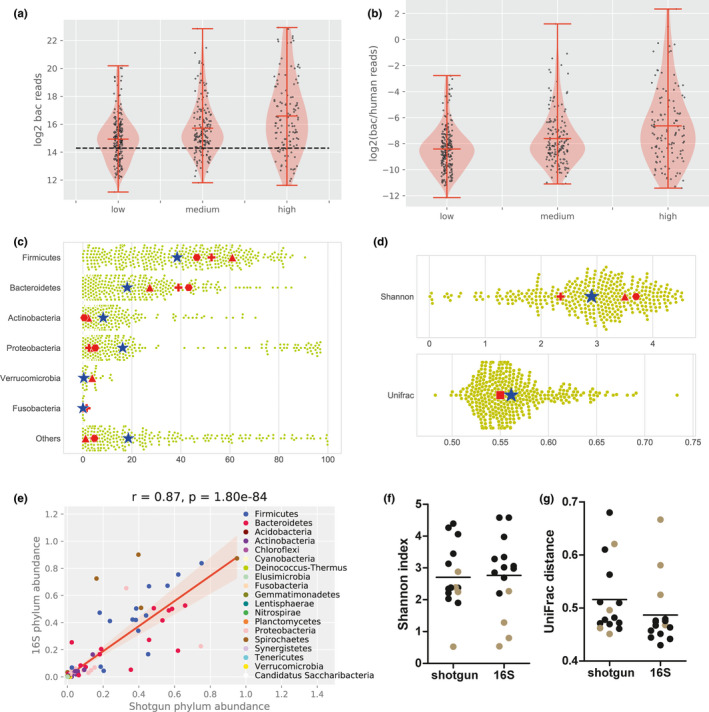
Shotgun metagenomic analysis of human colon tissue microbiomes. The number of bacterial reads (a) and the bacterial/human reads ratio (b) correlated to the visual estimated bacterial abundance assessed by microscopy. The black line represents the 20,000 read‐cutoff value. (c) The shotgun metagenomics of the clinical biopsies of our study was compared to 16S rRNA bacterial profiles from reported colon tissue microbiomes. The relative abundance of bacterial phyla is shown for study (dots) and the average is marked by a blue star. Averages of Diuric et al. (red triangle), Kiely et al. (red cross), and Watt et al. (red hexagon) are plotted in the graph. The Shannon diversity index and UniFrac distance are represented in (d), in which red square represents Momozawa et al. (e) Fifteen additional samples of follow‐up biopsies from the same patients from the BBC study were sequenced with both 16S rRNA and shotgun. Phylum abundance was strongly correlated between the methods (Pearson correlation 0.87, *p* < 0.001). The Shannon diversity index (f) and UniFrac distance (g) of the 15 double sequenced samples (shotgun and 16S) were plotted with the bar representing the mean. Four samples were dominated by spirochetes (brown)

Finally, we applied our optimized method to *in vivo* acquired colonic biopsies in the context of two prospective clinical studies (Supplementary Data S1: https://doi.org/10.5281/zenodo.4678214). The range of bacterial reads was 0.24%–40.51% vs 16.1–99.48% of human reads. Analysis showed that the number of bacterial reads was significantly associated with bacterial abundance determined by microscopy (KruskalResult, statistic = 38.310, *p* value = 4.8e−09) (Figure 4a). Bacterial abundance was scored on methacarn‐fixed paraffin‐embedded paired biopsies that were stained with fluorescent in situ hybridization (Probe EUB338 for most bacteria: 5’cy3‐ GCTGCCTCCCGTAGGAGT‐cy3'3) and scored by 2 or 3 independent observers by low, medium, or high bacterial abundance. The bacterial abundance score is also associated with the bacterial‐to‐human reads ratio (KruskalResult, statistic = 37.278, *p* value = 8.038e−09) (Figure 4b).

**FIGURE A6:**
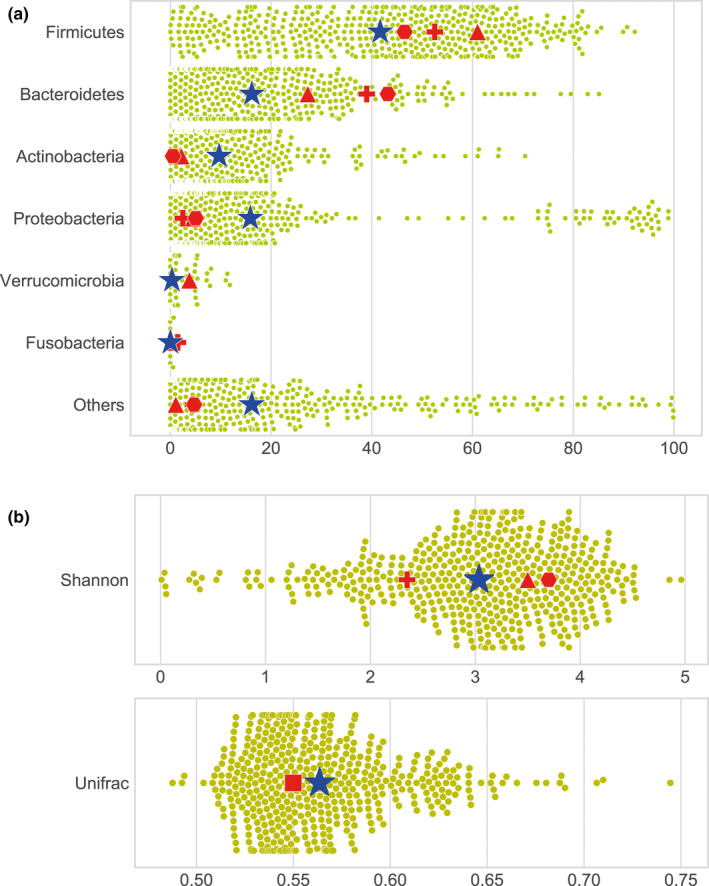
Unfiltered data of metagenomic bacterial profiles of clinical colon biopsies (without 20 000 bacterial reads as cutoff value). (a) Overall bacterial phyla profiles of all sequenced biopsies. (b) Shannon index and UniFrac distance of all sequenced biopsies

To be sure that we had sufficient reads for taxonomic classification, we analyzed samples with at least 20,000 classified bacterial reads (analysis of the complete dataset is also shown in Figure A6 in Appendix 2). Metagenomic analysis revealed that the most common phyla were *Firmicutes* (39.8%), *Bacteroidetes* (16.7%), *Actinobacteria* (9.3%), *Proteobacteria* (16.4%), *Verrocumicrobia* (0.2%) and others (17.5%) (Figure 4c). Thus far, shotgun metagenomics of microbiomes from tissue samples has been impeded by lack of bacterial DNA yield, so shotgun metagenomics has not been reported for colonic biopsies before. Here, we compared our data to samples sequenced by 16S rRNA sequencing (Table 1). We found a comparable distribution of bacterial phyla. Furthermore, the Shannon diversity of our study (2.9) was within range of other studies (2.4–3.7). Lastly, our study resulted in an average pairwise UniFrac distance of 0.56 (Figure 4d) which was similar to the UniFrac distance reported in Momozawa et al. (0.55).

**TABLE 1:** Microbiome profiles of human colon biopsies of our study (WGS) resemble those that have been previously published (16S rRNA)

	This study	Djuric et al.	Kiely et al.	Watt et al.	Momozawa et al.
Symbol Fig. 4	Blue star	Red triangle	Red cross	Red hexagon	Red square
*Firmicutes*	39.8	61	52.5	46.5	**–**
*Bacteroidetes*	16.7	27.3	39	43.2	**–**
*Actinobacteria*	9.3	2.2	**–**	0.5	**–**
*Proteobacteria*	16.4	4.5	2.5	5.1	**–**
*Verrucomicrobia*	0.2	3.8	**–**	**–**	**–**
*Fusobacteria*	0.0	0.1	1.5	**–**	**––**
*Others*	17.5	1.1	4.5	4.7	**–**
*Shannon index*	2.9	3.5	2.4	3.7	**–**
*I. Simpson index*	5.0	20.3	**–**	20	**–**
*UniFrac d*.	0.56	**–**	**–**	**–**	0.55

Note

We compared our microbiome profiles to those reported in Djuric et al., Kiely et al., Watt et al., and Momozawa et al. These results are represented with a symbol in Figure 4c + d. In this table, we report the relative abundances of bacterial phyla in percentage. Also, the Shannon index, inverse Simpson index (I. Simpson index), and UniFrac distance (UniFrac d.) are given when reported.

**FIGURE A7:**
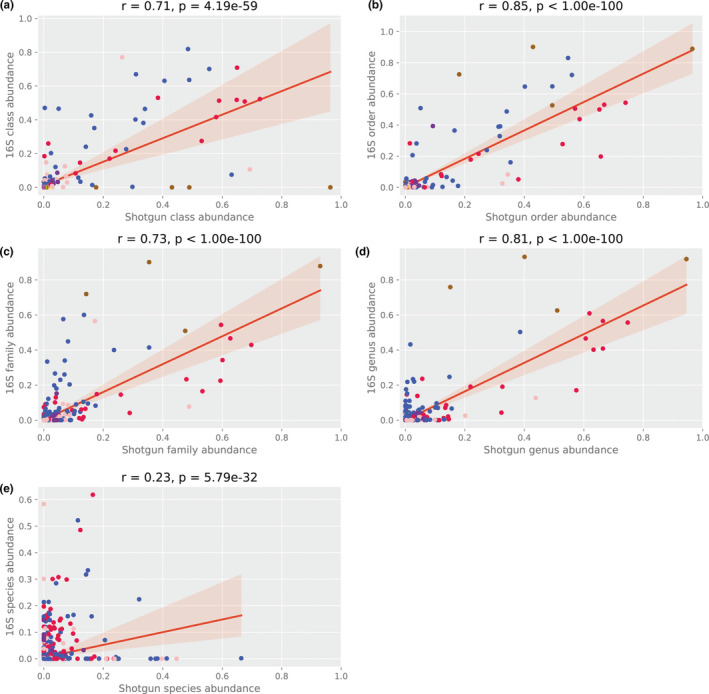
Correlation between 16S rRNA and shotgun sequencing of 15 clinical colon biopsies. 15 follow‐up biopsies of BBC study patients were sequenced with both 16S and shotgun sequencing and compared at class, order, family, genus, and species level (a–e, respectively)

Moreover, 15 additional biopsies acquired in the follow‐up from BBC study participants were sequenced with both 16S rRNA sequencing and shotgun metagenomics. These 15 biopsies have been selected because they had the highest DNA yield of a larger pool of follow‐up biopsy tissue isolates, thereby allowing sufficient yield for two sequencing methods of the same sample (Supplementary Data S2: https://doi.org/10.5281/zenodo.4678214). At phylum, class, order, family, and genus level, amplicon sequencing and shotgun highly correlated (Pearson: *r* = 0.87, *p* = 1.80e−84) (Figure 4e and Figure A7 in Appendix 2 for class to species level). Only at the species level, there was a low correlation. The Shannon diversity and UniFrac distance were not significantly different between the sequencing techniques (Figure 4f+g and Supplementary Data S2: https://doi.org/10.5281/zenodo.4678214). Notably, 4 of the 15 samples displayed spirochetosis, which could contribute to low Shannon diversity indices.

Although bacterial reads are sometimes still low, our optimized bacterial DNA isolation protocol (strategy 2) in combination with shotgun metagenomic sequencing was able to reproduce previously reported bacterial tissue profiles and direct comparison between shotgun metagenomics and 16S rRNA sequencing in samples sequenced with both methods shows high similarity. To our knowledge, this is the first time that colon tissue bacterial profiles have been reported with shotgun metagenomics.

## 4 DISCUSSION

Bacterial DNA isolation from tissues is complicated by large amounts of host DNA. While several strategies, protocols, and commercial kits have been developed to tackle this problem, so far none of these considered all elements that we considered important for the analysis of tissue bacteria. In this study, we developed a protocol, inspired by Molzym (2020) and Hasan et al. (2016), and the Human microbiome project (HMP) (Albuquerque et al., 2017), that enriched bacterial DNA through selective lysis of host DNA with 0.0125% saponin and subsequent DNAse treatment. This resulted in a bacterial DNA isolate in which the four most common phyla were represented, without inducing lysis of cultured bacterial cells or notably skewing bacterial composition in clinical biopsy samples. Of note, our strategy was shown to work also on fish gills and hence can be applied or tailored to other tissues similarly.

We started out testing the Ultra‐Deep Microbiome prep kit (Molzym, 2020) in combination with bead‐beating (strategy 1) because both methods perform well in microbiome research (Allali et al., 2015; Biesbroek et al., 2012; Knudsen et al., 2016; Marotz et al., 2018; Nelson et al., 2019; Yuan et al., 2012). The inclusion of bead‐beating enhanced isolation of all bacterial phyla, particularly *Actinobacteria* (Figure A1 in Appendix 2). Furthermore, we noticed that the detection of Gram‐negative bacteria could be improved by introducing a PBS wash, which we suspect to be caused by the premature lysis of Gram‐negative bacteria during the bacterial enrichment steps of this kit (Figure A3 in Appendix 2). This important limitation has been suggested before (Loonen et al., 2013).

The protocol that we set up (strategy 2) is an extended version of the protocol that we developed for processing fecal samples (Albuquerque et al., 2017). This protocol has been modified from the HMP protocol and includes an enzymatic lysis step with mutanolysin, heat shock, and bead‐beating. Our bead‐beating process has been optimized on fecal samples (Albuquerque et al., 2017). Importantly, fine‐tuning of bead‐beating speed and duration may be required for each specific bead‐beater. It has been questioned whether bead‐beating improves bacterial DNA isolation from tissues (Carbonero et al., 2011), because it may contribute to some level of DNA degradation (Carbonero et al., 2011; Moen et al., 2016). However, according to more recent studies, bead‐beating does not cause DNA shearing (Lim et al., 2018; Wagner Mackenzie et al., 2015) and results in the identification of extra species in tissue isolates (Yu et al., 2017). In our protocol and other studies, bead‐beating has proven to result in higher DNA yields (Carbonero et al., 2011), more efficient isolation of Gram‐positive bacteria (Biesbroek et al., 2012; Knudsen et al., 2016), a community structure that most closely resembles bacterial input (Yuan et al., 2012), and higher microbial diversity (Lim et al., 2018). Together, these findings suggest that bead‐beating should be included; however, it has to be performed with the right type of beads under the right conditions optimized in each laboratory.

Another important step in our protocol is the removal of human DNA from the isolate. Previous studies have reported human DNA removal (by qPCR) of roughly >90% in saliva and subgingival plaque samples with Molysis (Horz et al., 2010) and >90% in nasopharyngeal aspirate using TurboDNAse (Hasan et al., 2016). Our results showed a reduction of human DNA (by qPCR) of roughly 50% in tissue biopsies. To test whether TurboDNAse was working well, we tested whether TurboDNAse was able to remove DNA in DNA isolates. These results showed that TurboDNAse decreased the DNA concentration by 94%. We conclude that a large amount of human DNA is still inaccessible for DNAse‐mediated degradation during our protocol. Interestingly, the use of TurboDNAse without detergent also increased the bacterial‐to‐human DNA ratio. This was also observed before (Hasan et al., 2016). In the study of Hasan et al. (2016) the use of detergent resulted in a higher pathogen to host DNA ratio, while the attributable effect of detergent was not evident in our study (Figure 3c). We suspect that our results are impacted by the variety in biopsy size and hence the total amount of human DNA. A twofold decrease of human DNA signal was associated with a ~sevenfold increase in bacterial DNA signal in qPCR, indicating that human DNA content interferes strongly with the bacterial DNA signal. While it is evident that human DNA remains in the isolate, we have chosen to stick to a mild detergent (saponin 0.0125%) to prevent distortion of the microbiome profile, which may come at cost of complete human cell lysis.

While our protocol is optimized for our research goal (bacterial microbiome in two prospective clinical studies), it may require small adaptations for other research objectives. For example, since an important part of our protocol is a DNAse step in which bacterial DNA is still protected by cell‐wall separation, this DNA isolation protocol may not be optimal to detect bacteria without a cell wall, like mycoplasma. The study of these types of bacteria requires a different approach, of which antibody‐mediated filtering of bacterial DNA may still be an option. Small adaptations in the protocol may also improve the detection of certain bacterial subtypes, albeit at the cost of less efficient isolation of others. For example, Streptococci DNA yields may be even higher with more intense bead‐beating than in the current protocol. Noteworthy, we use saponin as a lysis agent. Since saponin targets cholesterol, it may also induce cell lysis of yeast (Francis et al., 2002) before DNAse treatment. The focus of this protocol is set on the isolation of the bacterial component of the microbiome, and we did not test how well it performs on yeasts. Hence, adaptations to have an accurate representation of yeast may be required. Importantly, our shotgun metagenomics sequencing detected archaea and viruses in all samples (Supplementary Data S1: https://doi.org/10.5281/zenodo.4678214).

Our shotgun metagenome sequencing results of 508 biopsies of 224 patients showed that we were able to produce bacterial profiles with Shannon diversity and UniFrac distance that is comparable to 16S rRNA sequencing data of colon tissues, indicating that this sequencing method can be used for tissue microbiome profiling. Nevertheless, small differences were observed between the bacterial composition of our study (shotgun) and three other studies (16S rRNA); we observed fewer *Bacteroidetes* and more *Actinobacteria*. Importantly, similar differences were found in another study comparing shotgun metagenomics with 16S rRNA in stool samples. Ranjan et al. reported fewer *Bacteroidetes* with shotgun metagenomics (14–21%) than with 16S rRNA sequencing (34%) and more *Actinobacteria* with shotgun metagenomics (4–7%) than with 16S rRNA sequencing (0.4%) (Ranjan et al., 2016). Hence, the differences observed between the colon tissue microbiomes of our and other studies may be caused by amplification biases.

While we have merged strategies from successful protocols and have created hand‐tailored steps in the protocol, further testing is necessary to confirm the preservation of microbial profiles in shotgun metagenomics vs amplicon sequencing in side‐by‐side comparisons. Our comparison of 15 samples with both shotgun metagenomics and 16S rRNA sequencing shows a high correlation of bacterial abundance between both methods on all taxonomic levels, except the species level, and a comparable Shannon diversity and UniFrac distance. More extensive analysis on genus and species level is required to firmly conclude that profiles are not skewed by the enrichment steps. Additionally, some experiments are of small size due to limited available material and the mock community only consisted of 4 different bacterial species. However, our protocol provides more insight than some currently commercially available kits and allows for the application of tissue shotgun metagenomics with comparable results to 16S rRNA sequencing based on available studies.

Taken together, here we show for the first time a protocol to be used for tissue shotgun metagenomics of colon biopsies that omits 16S rRNA amplification steps. Our protocol is mild enough to maintain isolation of Gram‐negative bacteria, while it also includes steps that facilitate isolation of sturdy bacteria like *Actinobacteria* and *Firmicutes*. Importantly, our protocol can also be tailored to isolate bacteria from other tissues, as has been demonstrated by its application to fish gills by an independent laboratory. In other words, our protocol can be immediately used for the analysis of stool and colon tissue samples, but may also serve as a foundation for isolation protocols for other study material. Moreover, while we chose shotgun metagenome sequencing, our protocol may also be used in combination with 16S rRNA amplicon sequencing. Thereby, our protocol applies to many different research settings where it facilitates the analysis of a wide spectrum of bacteria. This way our protocol may contribute to fundamental and clinical microbiome research, further illuminating the role of the microbiome in health and disease.

## CONFLICT OF INTEREST

None declared.

## AUTHOR CONTRIBUTION

**Carlijn E. Bruggeling:** Conceptualization (lead); Formal analysis (lead); Investigation (lead); Methodology (lead); Writing‐original draft (lead); Writing‐review & editing (lead). **Daniel R. Garza:** Data curation (lead); Formal analysis (equal); Investigation (equal); Methodology (lead); Resources (lead); Software (lead); Validation (equal); Writing‐original draft (equal); Writing‐review & editing (equal). **Soumia Achouiti:** Formal analysis (supporting); Investigation (supporting). **Wouter Mes:** Formal analysis (supporting); Investigation (supporting); Validation (supporting); Writing‐review & editing (supporting). **Bas E. Dutilh:** Conceptualization (equal); Funding acquisition (equal); Methodology (equal); Resources (equal); Software (equal); Supervision (equal); Writing‐review & editing (equal). **Annemarie Boleij:** Conceptualization (lead); Formal analysis (equal); Funding acquisition (lead); Investigation (equal); Methodology (equal); Project administration (lead); Supervision (lead); Validation (equal); Visualization (equal); Writing‐original draft (equal); Writing‐review & editing (lead).

## ETHICS STATEMENT

The research that has been conducted is following the Code of Ethics of the World Medical Association (Declaration of Helsinki). Clinical biopsies were acquired from the BBC study (NL57875.091.16) and the BaCo study (NL55930.091.16). Both studies were approved by the Internal Revenue Board CMO‐Arnhem Nijmegen (CMO 2016‐2616 and CMO 2016‐2818). All participants signed informed consent.

## Data Availability

All data generated are provided in full in the results and appendices of this paper. Supporting information including sequencing results is available at https://doi.org/10.5281/zenodo.4678214

## APPENDIX 1

**TABLE A1:** Schematic overview of experiments and material within this study. A short explanation for each action is provided below

Process	Action	Material	Figure
Pre‐work Testing bead‐beating (BB) and Molzym	1. Testing Molzym +BB	4 big biopsies (~5 mm) of patient nr.3 of resected colon	Figure A1 in Appendix 2
	2. Testing Molzym +BB with PBS wash	2 big biopsies of patient nr.4 of resected colon	Figure A2 (Biopsy and PBS wash isolated separately) in Appendix 2
	3. Testing Molzym enrichment on bacterial composition	4 mock communities	Figure A3 in Appendix 2
*Change to independent protocol with tweakable steps*; *change from DNA isolation strategy 1 to 2*.			
Protocol setup Detergent selection	4. Testing bacterial lysis under protocol conditions	Pure bacterial cultures	Figure 2a
	5. Testing which detergent causes the least difference to PBS	Total of 20 forceps (small) biopsies of patient nr. 1 + 2 of the resected colon (10 per patient)^a^	Figure 2b + Figure A4 + A5 in Appendix 2
	6. Test whether the selected detergent lyses human biopsies	6 big biopsies of patient nr. 7 of resected colon	Figure 2c
Protocol setup Confirm bacterial DNA enrichment	7. Test whether PBS wash should be included in DNAse treatment	12 big biopsies of patient nr. 5 + 6 of resected colons	Figure 3a+b
	8. Test which detergents result in the strongest bacterial DNA enrichment	Total of 20 forceps (small) biopsies of patient nr 1 + 2 of the resected colon (10 per patient)^a^	Figure 3c
Validation Sequencing results with our method	9. Evaluate whether the number of bacterial reads represents bacterial abundance by imaging	508 clinical *in vivo* acquired human biopsies of 224 patients	Figure 4a+b
	10. Observe which bacterial phyla are present		Figure 4c+d Figure A6 in Appendix 2
	11. Compare 16S with shotgun metagenomic sequencing of the same samples	15 follow‐up biopsies with high DNA yields	Figure 4e, f, and g Figure A7 in Appendix 2

^a^ Same material and experiment, but different aspects are shown in the figure.

### Table A1 description:

The goal of this paper was to set up a protocol for bacterial DNA isolation from human tissues which does not distort the bacterial profile, with attention to the following:

- With full tissue digestion to include all bacteria (residing close to or inside the tissue).
- Removes human DNA as much as possible.
- Does not lyse Gram‐negative bacteria too early in the process.
- Includes required steps for acquiring DNA from sturdy Gram‐positive bacteria.
- Creates reproducible bacterial profiles by sequencing without 16S rRNA amplification bias.

### Process description (Action # in table)

1. The Molzym DNA isolation kit was selected because this was a well‐reported strategy for bacterial DNA enrichment from human tissues. Our personalized bead‐beating (BB) protocol was inspired by HMP and previously optimized in our laboratories for feces. We tested Molzym with BB mainly to boost the isolation of sturdy Gram‐positive bacteria like *Actinobacteria*.
2. We compared the bacterial content of the PBS wash (PBS in which the biopsy was vortexed) with the bacterial content of the same washed biopsy. The PBS wash was not exposed to pre‐treatment and appeared to contain slightly more Gram‐negative bacteria, leading to suspicion that the lysis buffer of Molzym may lyse Gram‐negative bacteria too early in the protocol.
3. We tested Molzym with and without pre‐treatment on a mock community, which again raised the suspicion that Gram‐negative bacteria were lost due to pre‐treatment.
4. We decided to design our own lysis buffer. We tested which concentrations of saponin or Triton are safe to use on pure bacterial cultures.
5. We tested which concentrations of saponin or Triton would a cause shift in the relative abundance of most common phyla in resected biopsies.
6. We test whether saponin 0.0125% causes cell lysis by exposing human resection material to protocol conditions.
7. We tested whether the biopsy wash should be included in DNAse treatment (washing could break human cells and release human DNA in the supernatant).
8. We tested which detergent condition resulted in the best bacterial DNA enrichment.
9. We validated our protocol by performing shotgun metagenomic sequencing on in vivo acquired human biopsies of 2 prospective clinical studies. We tested whether the number of bacterial reads correlated with the bacterial abundance score that was rendered by imaging.
10. We evaluated whether common bacterial phyla of colon tissue microbiomes (reported previously in literature with 16S rRNA sequencing) were also represented in our samples that were isolated with our method (and processed with shotgun metagenomic sequencing).
11. We performed 16S rRNA and shotgun sequencing on 15 additional clinical biopsies (biopsies with high DNA yields to do both sequencing methods) and compared bacterial abundances, Shannon diversity, and UniFrac distance.

**TABLE A2:** Primers for qPCR.

Target	Forward primer	Reverse primer	References
Universal bacteria	926F: AAACTCAAAKGAATTGACGG	1062R: CTCACRRCACGAGCTGAC	Yang et al. (2015) & Bacchetti De Gregoris et al. (2011)
Firmicutes	928FirmF: TGAAACTYAAAGGAATTGACG	1040FirmR: ACCATGCACCACCTGTC	Bacchetti De Gregoris et al. (2011)
Bacteroidetes	Bac960F: GTTTAATTCGATGATACGCGAG	Bac1100R: TTAASCCGACACCTCACGG	Yang et al. (2015)
γ‐proteobacteria	1080γF: TCGTCAGCTCGTGTYGTGA	γ1202R: CGTAAGGGGCCATGATG	Bacchetti De Gregoris et al. (2011)
Actinobacteria	Act664: TGTAGCGGTGGAATGCGC	Act941R: AATTAAGCCACATGCTCCGCT	Yang et al. (2015)
Human KRAS	P696: AGGCCTGCTGAAAATGACTG	P488: TGGATCATATTCGTCCACAAAA	Silva et al. (2009)
Universal bacteria (used for fish gill experiment)	616F: AGAGTTTGATYMTGGCTCAG	Eub338IR: GCTGCCTCCCGTAGGAGT	Juretschko et al. (1998); Amann et al. (1990)
Zebrafish	LepA gen: GACTGCACACTGAAGGAATC	Lep A gen: GCACTGTCCTCTAGAAAAGC	Gorissen et al. (2009)

**TABLE A3:** Bacterial enrichment using saponin 0.0125% and TurboDNAse improves bacterial‐to‐fish DNA ratio in qPCR. DNA isolations were performed with and without DNAse treatment. Ct values are given in the upper part. In the lower part, the fold difference (FD) between the signal with and without DNA isolation is shown.

	With enrichment (Ct)		Without enrichment (Ct)	
	Bacterial signal	Host signal	Bacterial signal	Host signal
Fish gill isolate	32.08 35.47 35.94 29.13 27.95	30.45 31.02 31.58 28.25 30.17	33.01 33.22	23.47 22.96
Average	32.114	30.294	33.115	23.215

	ΔCt = Ct with – Ct without	
	FD Bacterial (2^−ΔCt^)	FD Host (2^−ΔCt^)
FD	2.001386775	0.0073962
1/FD	0.499653546	135.20456

## APPENDIX 3

### Protocol

#### Bacterial DNA isolation from tissue with bacterial enrichment and bead‐beating

**Reference: Optimized DNA isolation method for microbiome analysis of human tissues**. *Carlijn Bruggeling^1^*, *Daniel R. Garza^2^*, *Soumia Achouiti^1^*, *Wouter Mes^3^*, *Bas E. Dutilh^2^* ^,^ *^4^*, *Annemarie Boleij^1*^*

#### Goal

This protocol is optimized for bacterial DNA isolation from human colon tissue samples (~2–5 mm). During bacterial enrichment, the biopsy is vortexed in PBS to release bacteria from the biopsy. This supernatant (“biopsy wash”) is added back to the sample, after the rest of the biopsy is made into a cell suspension using proteinase K. The sample is treated with a soap to lyse human cells, which is combined with TurboDNAse treatment to digest external DNA. Subsequently, intact bacteria in the sample are sensitized to lysis using Mutanolysin and heat shock. Lastly, bead‐beating is used for mechanical lysis, which is followed by standard DNA isolation procedures.

Hereby, we provide a stepwise protocol.

#### Material

- PBS: Tris‐HCL (220/12257974/1110, Braun).
- Proteinase K (19133, Qiagen).
- Saponin 0.0125% (47036‐50G, Sigma‐Aldrich) in PBS, 0.2 µm filtered.
- TurboDNAse with 10× buffer (AM2239, Qiagen).
- Mutanolysin 10 KU in 2 ml ddH2O (SAE0092, Sigma‐Aldrich).
- DNeasy PowerLyzer PowerSoil kit (Qiagen).
- (previously known as MoBio Powerlyzer PowerSoil DNA isolation kit).
  - Bead solution.
  - Solution C1 to C6.
  - Beads (0.1 mm glass beads).
  - 3 sets of 2 mL collection tubes.
  - 1 set of spin filters.

#### Preparation:

Assure the following:

- Clean desk (with chloride).
- Centrifuge at 4°C.
- 70, 37, 65, and 95°C incubator.
- Ice bucket.
- Bead‐beater.

### Part 1: Bacterial enrichment

#### PBS wash and host tissue digestion

1. Prepare 2 sets of 1.5 ml Eppendorf tubes, of which 1 set with 500 µl PBS.

2. Put frozen biopsies in 500 µl PBS in a 1.5 ml tube (use a pipette tip).

3. Vortex tubes 5 min (speed 8/9).

Make PBS/Proteinase K mix

4. Transfer the supernatant (“biopsy wash”) to a new tube and keep it on ice.

5. If the biopsy is ~2 mm: add 197 µl of PBS and 3 µl of Proteinase K to the biopsy.

For larger biopsies: add 180 µl of PBS and 20 µl of Proteinase K to biopsy

6. Short spin down.

7. Incubate samples at 70°C, 400 rpm 15 min.

Set incubator to 37°C

8. Vortex shortly to assist tissue to fall apart.

9. Add 700 µl PBS to “biopsy wash” and add to matched biopsy (digested).

10. Spin at 10,000×*g* for 10 min 4°C.

Make Saponin/TurboDNAse/Buffer mix

11. Discard supernatant, save pellet

#### Host cell lysis and DNA digestion:

12. Add per biopsy 100 µl mix:

- 88 µl Saponin
- 10 µl buffer 10× Turbo DNAse buffer
- 2 µl TurboDNAse (2 Units/µl)

13. Resuspend by vortexing 15 s.

14. Short spin down.

15. Incubate at 37°C for 30 min 400 rpm.

16. Add 1.3 ml PBS.

17. Centrifuge at 10,000×*g*, 10 min at 4°C.

18. Discard supernatant by pipetting.

Make mutanolysin mix.

19. Add 1 mL PBS and resuspend the pellet by vortexing.

20. Centrifuge at 10,000×*g*, 10 min at 4°C.

21. Discard supernatant by pipetting.

22. Store pellets at −20°C or go to step 23.

### Part 2: Bead‐beating protocol

#### Bead‐beating preparation:

23. Add 180 µl of Bead solution +20 µl of mutanolysin per sample.

24. Resuspend by vortexing.

25. Incubate at 37°C for 60 min 400 rpm.

Set up the heater at 6°C

26. Put tubes in the incubator at 400 rpm:

65°C for 10 min,

heat‐up to 95°C (7 min)

95°C for 10 min

27. Cool down to room temperature and spin down shortly.

#### Bead‐beating:

28. Add 550 µl of Power bead solution to the sample.

29. Vortex tubes for 30 to 40 s.

30. Add mixture to bead tubes.

31. Add 60 µl of solution C1 (first solution of DNeasy isolation kit).

Prevent cooling the sample, but bring ice for the following step.

32. Bead‐beat with the MagNA Lyser:

- 6400 rpm for 30 s
- On ice for 30 s
- 6400 rpm for 30 s

Keep samples on ice

​

#### Bacterial DNA extraction

33. Centrifuge at 10,000×*g* for 2 min.

34. Transfer supernatant to a new set of collection tubes.

*Keep a maximum total volume of 500 µl.

35. Add 250 µl of solution C2, Vortex for 5 s, incubate on ice for 5 min.

36. Centrifuge at 10,000×*g* for 1 min.

37. Transfer up to 600–800 µl to the 2 ml collection tubes.

38. Add 200 µl of solution C3, vortex briefly, then place on ice for 5 min.

39. Centrifuge at 10,000×*g* for 1 min.

40. Transfer up to 750 µl of supernatant to the 2 ml collection tubes.

41. Add as much as possible without disturbing the pellet (~850 µl).

42. Shake solution C4, add 1.2 ml (2 × 600 µl), Vortex for 5 s.

43. Add as much as possible, ~1 ml, avoid that it is so full that it splashes.

44. Load approximately 675 µl onto a spin filter, centrifuge at 10,000×*g* for 1 min, Discard the flow (do this 3 until the sample is finished).

45. Add 500 µl of solution C5, centrifuge at 10,000×*g* for 30 s.

46. Discard the flow‐through.

47. Centrifuge at 10,000×*g* for 1 min.

48. Carefully place a spin filter in a new set of collection tubes.

49. Add 50 µl of solution C6 to the center of the membrane.

50. Centrifuge at 10,000×*g* for 30 s.

51. Discard the Spin Filter.

52. Store the extracted DNA at −80°C.

## APPENDIX 4

### CTAB extraction

#### Buffer

100 mM Tris‐HCl.

100 mM Na‐EDTA.

1.5 M NaCl.

2% CTAB.

0.05 mg/ml proteinase K.

#### Material

10% SDS.

Chloroform:isoamyl alcohol (24:1).

Isopropanol.

Phenol:chloroform:isoamyl alcohol (25:24:1).

3 M Na‐acetate.

100% EtOH.

70% EtOH.

Autoclaved milliQ H_2_O.

#### CTAB extraction of genomic DNA from de‐enriched zebrafish gills

- After the digestion of gill samples with DNase, resuspend washed pellet in 100 µl CTAB extraction buffer and incubate at 37°C for 30 min, mixing every 5 min by inverting the tubes.
- Add 25 µl 10% SDS to sample, mix well and incubate for 1 hr at 65°C. Mix every 5 min by inverting the tubes.
- Add 125 µl chloroform:isoamyl alcohol and mix thoroughly for 20 s.
- Centrifuge samples at max. speed for 15 min.
- Transfer aqueous phase into clean tubes, discard waste into a container in the fumehood.
- Add 0.6 volumes of isopropanol to samples and incubate overnight at −20°C.
- Centrifuge samples at max. speed for 15 min.
- Pour off isopropanol carefully (don't lose pellet).
- Wash pellet with 500 µl 70% EtOH, centrifuge 10 min. at maximum *g*.
- Pour off ethanol carefully.
- Leave tubes open for 5 min to evaporate the remaining ethanol.
- Resuspend pellet in 200 µl autoclaved milliQ.

#### RNase treatment of DNA extractions

- Add 1 µl (10 mg/ml) RNase A to samples, incubate at 37°C for 30 min.
- Add 200 µl phenol:chloroform:isoamyl alcohol, mix thoroughly for 20 s.
- Centrifuge 15 min at maximum speed.
- Transfer aqueous phase into a new tube, discard phenol waste into a container in the fumehood.
- Add 2 volumes of 100% EtOH and 0.1 volume of NaAc, mix by inverting the tube.
- Incubate at −20°C for 1 hr.
- Pellet DNA by centrifuging for 20 min at maximum speed.
- Wash pellet with 500 µl 70% EtOH, centrifuge 10 min at maximum speed.
- Pour off ethanol carefully, spin down the rest of the ethanol by short centrifugation.
- Remove residual ethanol by pipetting, without disturbing the pellet.
- Dry pellet until all ethanol is evaporated.
- Resuspend pellet in 50 µl autoclaved milliQ water.

#### PCR

**Table:**

**qPCR program**		
3:00	96°C	1×
0:15	96°C	40×
0:20	58°C	
0:30	72°C	
2:00	72°C	1×

**Table:**

qPCR mix	
SYBR mix 2×	10 µl
Forward (10 μM)	0.6 µl
Reverse (10 μM)	0.6 µl
H_2_O	… µl (upto 20 µl)
DNA	5 ng

## References

[mbo31191-bib-0001] Allali, I. , Delgado, S. , Marron, P. I. , Astudillo, A. , Yeh, J. J. , Ghazal, H. , Amzazi, S. , Keku, T. , & Azcarate‐Peril, M. A. (2015). Gut microbiome compositional and functional differences between tumor and non‐tumor adjacent tissues from cohorts from the US and Spain. Gut Microbes, 6(3), 161–172. 10.1080/19490976.2015.1039223. 25875428 PMC4615176

[mbo31191-bib-0002] Amann, R. I. , Binder, B. J. , Olson, R. J. , Chisholm, S. W. , Devereux, R. , & Stahl, D. A. (1990). Combination of 16S rRNA‐targeted oligonucleotide probes with flow cytometry for analyzing mixed microbial populations. Applied and Environment Microbiology, 56(6), 1919–1925. 10.1128/aem.56.6.1919-1925.1990 PMC184531 2200342

[mbo31191-bib-0003] Bacchetti De Gregoris, T. , Aldred, N. , Clare, A. S. , & Burgess, J. G. (2011). Improvement of phylum‐ and class‐specific primers for real‐time PCR quantification of bacterial taxa. Journal of Microbiol Methods, 86(3), 351–356. 10.1016/j.mimet.2011.06.010. 21704084

[mbo31191-bib-0004] Bajaj, J. S. , Hylemon, P. B. , Ridlon, J. M. , Heuman, D. M. , Daita, K. , White, M. B. , Monteith, P. , Noble, N. A. , Sikaroodi, M. , & Gillevet, P. M. (2012). Colonic mucosal microbiome differs from stool microbiome in cirrhosis and hepatic encephalopathy and is linked to cognition and inflammation. American Journal of Physiology. Gastrointestinal and Liver Physiology, 303(6), G675–685. 10.1152/ajpgi.00152.2012. 22821944 PMC3468538

[mbo31191-bib-0006] Biesbroek, G. , Sanders, E. A. M. , Roeselers, G. , Wang, X. , Caspers, M. P. M. , Trzciński, K. , Bogaert, D. , & Keijser, B. J. F. (2012). Deep sequencing analyses of low density microbial communities: working at the boundary of accurate microbiota detection. PLoS One, 7(3), e32942. 10.1371/journal.pone.0032942. 22412957 PMC3295791

[mbo31191-bib-0007] Bjerre, R. D. , Hugerth, L. W. , Boulund, F. , Seifert, M. , Johansen, J. D. , & Engstrand, L. (2019). Effects of sampling strategy and DNA extraction on human skin microbiome investigations. Scientific Reports, 9(1), 17287. 10.1038/s41598-019-53599-z. 31754146 PMC6872721

[mbo31191-bib-0008] Bokulich, N. A. , Subramanian, S. , Faith, J. J. , Gevers, D. , Gordon, J. I. , Knight, R. , Mills, D. A. , & Caporaso, J. G. (2013). Quality‐filtering vastly improves diversity estimates from Illumina amplicon sequencing. Nature Methods, 10(1), 57–59. 10.1038/nmeth.2276. 23202435 PMC3531572

[mbo31191-bib-0009] Caporaso, J. G. , Kuczynski, J. , Stombaugh, J. , Bittinger, K. , Bushman, F. D. , Costello, E. K. , Fierer, N. , Peña, A. G. , Goodrich, J. K. , Gordon, J. I. , Huttley, G. A. , Kelley, S. T. , Knights, D. , Koenig, J. E. , Ley, R. E. , Lozupone, C. A. , McDonald, D. , Muegge, B. D. , Pirrung, M. , … Knight, R. (2010). QIIME allows analysis of high‐throughput community sequencing data. Nature Methods, 7(5), 335–336. 10.1038/nmeth.f.303. 20383131 PMC3156573

[mbo31191-bib-0010] Carbonero, F. , Nava, G. M. , Benefiel, A. C. , Greenberg, E. , & Gaskins, H. R. (2011). Microbial DNA extraction from intestinal biopsies is improved by avoiding mechanical cell disruption. Journal of Microbiol Methods, 87(1), 125–127. 10.1016/j.mimet.2011.07.014. PMC3168962 21820015

[mbo31191-bib-0011] Cattonaro, F. , Spadotto, A. , Radovic, S. , & Marroni, F. (2018). Do you cov me? Effect of coverage reduction on metagenome shotgun sequencing studies. F1000Res, 7, 1767. 10.12688/f1000research.16804.4. 32185014 PMC7059852

[mbo31191-bib-0012] Couto Furtado Albuquerque, M. , van Herwaarden, Y. , Kortman, G. A. M. , Dutilh, B. E. , Bisseling, T. , & Boleij, A. (2017). Preservation of bacterial DNA in 10‐year‐old guaiac FOBT cards and FIT tubes. Journal of Clinical Pathology, 70(11), 994–996. 10.1136/jclinpath-2017-204592. 28830908 PMC5749348

[mbo31191-bib-0013] de Boer, R. , Peters, R. , Gierveld, S. , Schuurman, T. , Kooistra‐Smid, M. , & Savelkoul, P. (2010). Improved detection of microbial DNA after bead‐beating before DNA isolation. Journal of Microbiol Methods, 80(2), 209–211. 10.1016/j.mimet.2009.11.009. 19995580

[mbo31191-bib-0014] Djuric, Z. , Bassis, C. M. , Plegue, M. A. , Sen, A. , Turgeon, D. K. , Herman, K. , & Ruffin, M. T. (2019). Increases in Colonic Bacterial Diversity after omega‐3 Fatty Acid Supplementation Predict Decreased Colonic Prostaglandin E2 Concentrations in Healthy Adults. Journal of Nutrition, 149(7), 1170–1179. 10.1093/jn/nxy255. PMC6602899 31051496

[mbo31191-bib-0015] Edgar, R. C. (2013). UPARSE: highly accurate OTU sequences from microbial amplicon reads. Nature Methods, 10(10), 996–998. 10.1038/nmeth.2604. 23955772

[mbo31191-bib-0016] Edgar, R. C. , Haas, B. J. , Clemente, J. C. , Quince, C. , & Knight, R. (2011). UCHIME improves sensitivity and speed of chimera detection. Bioinformatics, 27(16), 2194–2200. 10.1093/bioinformatics/btr381. 21700674 PMC3150044

[mbo31191-bib-0017] Francis, G. , Kerem, Z. , Makkar, H. P. , & Becker, K. (2002). The biological action of saponins in animal systems: a review. British Journal of Nutrition, 88(6), 587–605. 10.1079/bjn2002725. 12493081

[mbo31191-bib-0018] Gevers, D. , Knight, R. , Petrosino, J. F. , Huang, K. , McGuire, A. L. , Birren, B. W. , Nelson, K. E. , White, O. , Methé, B. A. , & Huttenhower, C. (2012). The Human Microbiome Project: a community resource for the healthy human microbiome. PLoS Biology, 10(8), e1001377. 10.1371/journal.pbio.1001377. 22904687 PMC3419203

[mbo31191-bib-0019] Gorissen, M. , Bernier, N. J. , Nabuurs, S. B. , Flik, G. , & Huising, M. O. (2009). Two divergent leptin paralogues in zebrafish (*Danio rerio*) that originate early in teleostean evolution. Journal of Endocrinology, 201(3), 329–339. 10.1677/joe-09-0034. 19293295

[mbo31191-bib-0020] Hasan, M. R. , Rawat, A. , Tang, P. , Jithesh, P. V. , Thomas, E. , Tan, R. , & Tilley, P. (2016). Depletion of human DNA in spiked clinical specimens for improvement of sensitivity of pathogen detection by next‐generation sequencing. Journal of Clinical Microbiology, 54(4), 919–927. 10.1128/JCM.03050-15. 26763966 PMC4809942

[mbo31191-bib-0021] Horz, H. P. , Scheer, S. , Vianna, M. E. , & Conrads, G. (2010). New methods for selective isolation of bacterial DNA from human clinical specimens. Anaerobe, 16(1), 47–53. 10.1016/j.anaerobe.2009.04.009. 19463963

[mbo31191-bib-0022] Juretschko, S. , Timmermann, G. , Schmid, M. , Schleifer, K. H. , Pommerening‐Röser, A. , Koops, H. P. , & Wagner, M. (1998). Combined molecular and conventional analyses of nitrifying bacterium diversity in activated sludge: Nitrosococcus mobilis and Nitrospira‐like bacteria as dominant populations. Applied and Environment Microbiology, 64(8), 3042–3051. 10.1128/aem.64.8.3042-3051.1998 PMC106813 9687471

[mbo31191-bib-0023] Kiely, C. J. , Pavli, P. , & O'Brien, C. L. (2018). The role of inflammation in temporal shifts in the inflammatory bowel disease mucosal microbiome. Gut Microbes, 9(6), 477–485. 10.1080/19490976.2018.1448742. 29543557 PMC6287691

[mbo31191-bib-0024] Knudsen, B. E. , Bergmark, L. , Munk, P. , Lukjancenko, O. , Prieme, A. , Aarestrup, F. M. , & Pamp, S. J. (2016). Impact of Sample Type and DNA Isolation Procedure on Genomic Inference of Microbiome Composition. mSystems, 1(5). e00095–16. 10.1128/mSystems.00095-16 27822556 PMC5080404

[mbo31191-bib-0025] Lim, M. Y. , Song, E. J. , Kim, S. H. , Lee, J. , & Nam, Y. D. (2018). Comparison of DNA extraction methods for human gut microbial community profiling. Systematic and Applied Microbiology, 41(2), 151–157. 10.1016/j.syapm.2017.11.008. 29305057

[mbo31191-bib-0026] Loonen, A. J. M. , Bos, M. P. , van Meerbergen, B. , Neerken, S. , Catsburg, A. , Dobbelaer, I. , Penterman, R. , Maertens, G. , van de Wiel, P. , Savelkoul, P. , & van den Brule, A. J. C. (2013). Comparison of pathogen DNA isolation methods from large volumes of whole blood to improve molecular diagnosis of bloodstream infections. PLoS One, 8(8), e72349. 10.1371/journal.pone.0072349. 23977288 PMC3744477

[mbo31191-bib-0027] Louca, S. , Doebeli, M. , & Parfrey, L. W. (2018). Correcting for 16S rRNA gene copy numbers in microbiome surveys remains an unsolved problem. Microbiome, 6(1), 41. 10.1186/s40168-018-0420-9. 29482646 PMC5828423

[mbo31191-bib-0028] Lozupone, C. , & Knight, R. (2005). UniFrac: a new phylogenetic method for comparing microbial communities. Applied and Environment Microbiology, 71(12), 8228–8235. 10.1128/aem.71.12.8228-8235.2005. PMC1317376 16332807

[mbo31191-bib-0029] Lynch, S. V. , & Pedersen, O. (2016). The Human Intestinal Microbiome in Health and Disease. New England Journal of Medicine, 375(24), 2369–2379. 10.1056/NEJMra1600266. 27974040

[mbo31191-bib-0030] Magoc, T. , & Salzberg, S. L. (2011). FLASH: fast length adjustment of short reads to improve genome assemblies. Bioinformatics, 27(21), 2957–2963. 10.1093/bioinformatics/btr507. 21903629 PMC3198573

[mbo31191-bib-0031] Marotz, C. A. , Sanders, J. G. , Zuniga, C. , Zaramela, L. S. , Knight, R. , & Zengler, K. (2018). Improving saliva shotgun metagenomics by chemical host DNA depletion. Microbiome, 6(1), 42. 10.1186/s40168-018-0426-3. 29482639 PMC5827986

[mbo31191-bib-0032] Moen, A. E. , Tannaes, T. M. , Vatn, S. , Ricanek, P. , Vatn, M. H. , & Jahnsen, J. (2016). Simultaneous purification of DNA and RNA from microbiota in a single colonic mucosal biopsy. BMC Research Notes, 9, 328. 10.1186/s13104-016-2110-7. 27352784 PMC4924232

[mbo31191-bib-0033] Molzym . https://www.molzym.com/next‐generation‐sequencing/ultra‐deep‐microbiome‐prep

[mbo31191-bib-0034] Momozawa, Y. , Deffontaine, V. , Louis, E. , & Medrano, J. F. (2011). Characterization of bacteria in biopsies of colon and stools by high throughput sequencing of the V2 region of bacterial 16S rRNA gene in human. PLoS One, 6(2), e16952. 10.1371/journal.pone.0016952. 21347324 PMC3037395

[mbo31191-bib-0035] Nelson, M. T. , Pope, C. E. , Marsh, R. L. , Wolter, D. J. , Weiss, E. J. , Hager, K. R. , Vo, A. T. , Brittnacher, M. J. , Radey, M. C. , Hayden, H. S. , Eng, A. , Miller, S. I. , Borenstein, E. , & Hoffman, L. R. (2019). Human and Extracellular DNA Depletion for Metagenomic Analysis of Complex Clinical Infection Samples Yields Optimized Viable Microbiome Profiles. Cell Reports, 26(8), 2227–2240. 10.1016/j.celrep.2019.01.091. 30784601 PMC6435281

[mbo31191-bib-0036] Quast, C. , Pruesse, E. , Yilmaz, P. , Gerken, J. , Schweer, T. , Yarza, P. , Peplies, J. , & Glöckner, F. O. (2013). The SILVA ribosomal RNA gene database project: improved data processing and web‐based tools. Nucleic Acids Research, 41(Database issue), D590–596. 10.1093/nar/gks1219. 23193283 PMC3531112

[mbo31191-bib-0037] Ranjan, R. , Rani, A. , Metwally, A. , McGee, H. S. , & Perkins, D. L. (2016). Analysis of the microbiome: Advantages of whole genome shotgun versus 16S amplicon sequencing. Biochemical and Biophysical Research Communications, 469(4), 967–977. 10.1016/j.bbrc.2015.12.083. 26718401 PMC4830092

[mbo31191-bib-0038] Saffarian, A. , Mulet, C. , Regnault, B. , Amiot, A. , Tran‐Van‐Nhieu, J. , Ravel, J. , Sobhani, I. , Sansonetti, P. J. , & Pédron, T. (2019). Crypt‐ and Mucosa‐associated core microbiotas in humans and their alteration in colon cancer patients. MBio, 10(4). e01315–19. 10.1128/mBio.01315-19. 31311881 PMC6635529

[mbo31191-bib-0005] Silva, F. P. G. , Almeida, I. , Morolli, B. , Brouwer‐Mandema, G. , Wessels, H. , Vossen, R. , Vrieling, H. , Marijt, E. W.A. , Valk, P. J.M. , Kluin‐Nelemans, H. C. , Sperr, W. R. , Ludwig W.‐D. , & Giphart‐Gassler, M. (2009). Genome wide molecular analysis of minimally differentiated acute myeloid leukemia. Haematologica, 94,(11), 1546–1554. 10.3324/haematol.2009.009324. 19773259 PMC2770965

[mbo31191-bib-0039] Taddese, R. , Garza, D. R. , Ruiter, L. N. , de Jonge, M. I. , Belzer, C. , Aalvink, S. , & Boleij, A. (2019). Growth rate alterations of human colorectal cancer cells by 157 gut bacteria. bioRxiv, 2019.2012.2014.876367. 10.1101/2019.12.14.876367 PMC7524400 32915102

[mbo31191-bib-0040] Thoendel, M. , Jeraldo, P. R. , Greenwood‐Quaintance, K. E. , Yao, J. Z. , Chia, N. , Hanssen, A. D. , Abdel, M. P. , & Patel, R. (2016). Comparison of microbial DNA enrichment tools for metagenomic whole genome sequencing. Journal of Microbiol Methods, 127, 141–145. 10.1016/j.mimet.2016.05.022. PMC5752108 27237775

[mbo31191-bib-0041] von Meijenfeldt, F. A. B. , Arkhipova, K. , Cambuy, D. D. , Coutinho, F. H. , & Dutilh, B. E. (2019). Robust taxonomic classification of uncharted microbial sequences and bins with CAT and BAT. Genome Biology, 20(1), 217. 10.1186/s13059-019-1817-x. 31640809 PMC6805573

[mbo31191-bib-0042] Wagner Mackenzie, B. , Waite, D. W. , & Taylor, M. W. (2015). Evaluating variation in human gut microbiota profiles due to DNA extraction method and inter‐subject differences. Frontiers in Microbiology, 6, 130. 10.3389/fmicb.2015.00130. 25741335 PMC4332372

[mbo31191-bib-0043] Wang, Q. , Garrity, G. M. , Tiedje, J. M. , & Cole, J. R. (2007). Naive Bayesian classifier for rapid assignment of rRNA sequences into the new bacterial taxonomy. Applied and Environment Microbiology, 73(16), 5261–5267. 10.1128/AEM.00062-07. PMC1950982 17586664

[mbo31191-bib-0044] Watt, E. , Gemmell, M. R. , Berry, S. , Glaire, M. , Farquharson, F. , Louis, P. , Murray, G. I. , El‐Omar, E. , & Hold, G. L. (2016). Extending colonic mucosal microbiome analysis‐assessment of colonic lavage as a proxy for endoscopic colonic biopsies. Microbiome, 4(1), 61. 10.1186/s40168-016-0207-9. 27884202 PMC5123352

[mbo31191-bib-0045] Wesolowska‐Andersen, A. , Bahl, M. I. , Carvalho, V. , Kristiansen, K. , Sicheritz‐Ponten, T. , Gupta, R. , & Licht, T. R. (2014). Choice of bacterial DNA extraction method from fecal material influences community structure as evaluated by metagenomic analysis. Microbiome, 2, 19. 10.1186/2049-2618-2-19. 24949196 PMC4063427

[mbo31191-bib-0046] Yang, Y.‐W. , Chen, M.‐K. , Yang, B.‐Y. , Huang, X.‐J. , Zhang, X.‐R. , He, L.‐Q. , Zhang, J. , & Hua, Z.‐C. (2015). Use of 16S rRNA Gene‐Targeted Group‐Specific Primers for Real‐Time PCR Analysis of Predominant Bacteria in Mouse Feces. Applied and Environment Microbiology, 81(19), 6749–6756. 10.1128/aem.01906-15. PMC4561689 26187967

[mbo31191-bib-0047] Yu, G. , Hu, N. , Wang, L. , Wang, C. , Han, X.‐Y. , Humphry, M. , Ravel, J. , Abnet, C. C. , Taylor, P. R. , & Goldstein, A. M. (2017). Gastric microbiota features associated with cancer risk factors and clinical outcomes: A pilot study in gastric cardia cancer patients from Shanxi, China. International Journal of Cancer, 141(1), 45–51. 10.1002/ijc.30700. 28319273 PMC5839466

[mbo31191-bib-0048] Yuan, S. , Cohen, D. B. , Ravel, J. , Abdo, Z. , & Forney, L. J. (2012). Evaluation of methods for the extraction and purification of DNA from the human microbiome. PLoS One, 7(3), e33865. 10.1371/journal.pone.0033865. 22457796 PMC3311548

[mbo31191-bib-0049] Zeller, G. , Tap, J. , Voigt, A. Y. , Sunagawa, S. , Kultima, J. R. , Costea, P. I. , Amiot, A. , Böhm, J. , Brunetti, F. , Habermann, N. , Hercog, R. , Koch, M. , Luciani, A. , Mende, D. R. , Schneider, M. A. , Schrotz‐King, P. , Tournigand, C. , Tran Van Nhieu, J. , Yamada, T. , … Bork, P. (2014). Potential of fecal microbiota for early‐stage detection of colorectal cancer. Molecular Systems Biology, 10, 766. 10.15252/msb.20145645. 25432777 PMC4299606

